# Modes of Action of ADP-Ribosylated Elongation Factor 2 in Inhibiting the Polypeptide Elongation Cycle: A Modeling Study

**DOI:** 10.1371/journal.pone.0066446

**Published:** 2013-07-08

**Authors:** Kevin C. Chen, Honglin Xie, Yujie Cai

**Affiliations:** Multidisciplinary Research Center, Shantou University, Guangdong, China; Beijing Institute of Genomics, China

## Abstract

Despite the fact that ADP-ribosylation of eukaryotic elongation factor 2 (EF2) leads to inhibition of protein synthesis, the mechanism by which ADP-ribosylated EF2 (ADPR•EF2) causes this inhibition remains controversial. Here, we applied modeling approaches to investigate the consequences of various modes of ADPR•EF2 inhibitory actions on the two coupled processes, the polypeptide chain elongation and ADP-ribosylation of EF2. Modeling of experimental data indicates that ADPR•EF2 fully blocks the late-phase translocation of tRNAs; but the impairment in the translocation upstream process, mainly the GTP-dependent factor binding with the pretranslocation ribosome and/or the guanine nucleotide exchange in EF2, is responsible for the overall inhibition kinetics. The reduced ADPR•EF2-ribosome association spares the ribosome to bind and shield native EF2 against toxin attack, thereby deferring the inhibition of protein synthesis inhibition and inactivation of EF2. Minimum association with the ribosome also keeps ADPR•EF2 in an accessible state for toxins to catalyze the reverse reaction when nicotinamide becomes available. Our work underscores the importance of unveiling the interactions between ADPR•EF2 and the ribosome, and argues against that toxins inhibit protein synthesis through converting native EF2 to a competitive inhibitor to actively disable the ribosome.

## Introduction

The functional structures of ADP-ribosylating toxins such as diphtheria toxin [1], [2] and pseudomonas exotoxin [3] contain two interlinked moieties. One moiety possesses adenosine diphosphate-ribosyl (ADPR) transferase activity; the other is responsible for binding with the cell membrane receptors and for subsequent transmembrane transport of the ADPR-transferasing fragment to the cytosol. Once in the cytosol, the toxin fragments catalytically transfer an ADP-ribose moiety in nicotinamide adenine dinucleotide (NAD^+^) to a posttranslationally modified histidine residue, termed diphthamide [1]–[3], in the eukaryotic elongation factor 2 (EF2). ADP-ribosylated EF2 (ADPR•EF2) is inactive in catalyzing the translocation of peptidyl-tRNAs on the ribosome, thus preventing nascent protein synthesis. While a growing body of knowledge has accumulated on the role of EF2 and EF-G (the prokaryotic homolog of EF2) in the translocation of tRNAs on the ribosome [4]–[10], the specific events abolished by ADPR•EF2 that culminate to the inhibition of protein synthesis remain elusive. Past studies [11], [12] observed that ADPR•EF2 in the presence of GTP fails to bring the 80S pretranslocation (PRE) ribosome to a puromycin-reactive posttranslocated (POST) state. Puromycin is an aminonucleoside antibiotic that mimics the 3′ end of the aminoacyl-tRNA, and hence binds only the POST-state ribosome to induce peptidyl transfer of the growing polypeptide chian at the P site. Although the puromycin unreactivity unequivocably establishes that translocation of the peptidyl-tRNA on the PRE ribosome is halted in the presence of ADPR•EF2, the mechanisms underpinning the observed protein synthesis inhibition are not well agreed upon. A number of works reported that ADPR•EF2 reduced GTPase activity [13], [14], reduced binding affinities to specific/non-specific rRNAs [15], [16], to the 80S ribosome [14], [17]–[20], and/or to GTP [20], [21]. These findings suggest that inhibition of nascent protein syntheses may result from ADPR•EF2 abolishing the upstream event(s) before translocation. Countering evidence, however, advocated that ADPR•EF2 bound competitively against native EF2 for the PRE ribosome [11], [22]–[25] or GTP [14], [18], [25]–[27], implying that ADPR•EF2 does not compromise the upstream processes of translocation but directly inhibits a late-phase tRNA translocation [7], [25] to disrupt protein synthesis.

To date, the dispute remains concerning the main elongation events compromised by ADPR•EF2 that lead to inhibition of protein synthesis. As such, the kinetics of inhibition exerted by toxin action on nascent peptide incorporation and the coupled ADP-ribosylation reaction are less clear. Here, we aim to investigate the correlations between modes of ADPR•EF2 action and the resulting inhibition kinetics via computer simulations, believing that comparing simulations to appropriate experimental data should allow us to infer the plausible elongation event(s) impaired by ADPR•EF2. We emphasize that this approach does not constitute as a proof of the proposed inhibition mechanism; it could only rule out some possibilities because their simulations do not fit the data. Based on simulations of cell-free protein synthesis data, we propose that ADP-ribosylation of EF2, in addition to fully blocking the late-phase translocation, also exerts an incomplete but substantial impairment to the apparent binding of ADPR•EF2•GTP with the PRE ribosome or to the exchange of GTP in ADPR•EF2•GDP.

## Materials and Methods

### The Model for Elongation Cycle

**Figure 1:**
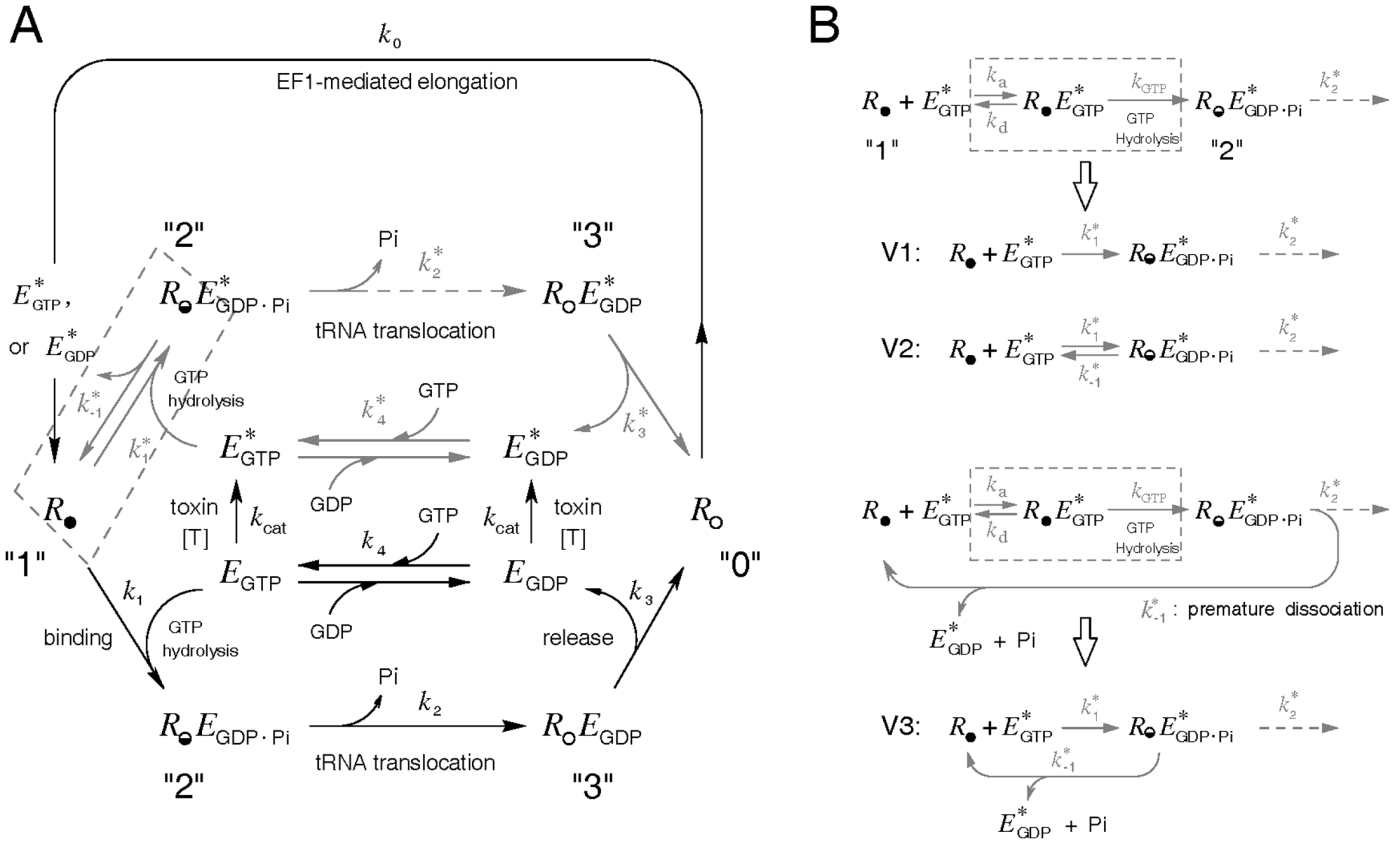
Schematics for the polypeptide chain elongation cycle. (*A*) The ribosome is divided into four phases: the A-sites vacated POST phase *R*_O_ ready to receive a cognate aminoacyl-tRNA (phase “0”), the PRE phase 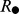 bearing a growing peptidyl-tRNA in the A site (phase “1”), the PRE ribosomal complex 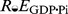 in transition of translocation (phase “2”), and the POST ribosomal complex *R*_O_ *E*_GDP_ (phase “3”). Phases are interconnected by four reactions representing the EF1α-initiated peptide elongation (*k*_0_), the combined factor binding and GTP hydrolysis (*k*_1_), the EF2-mediated tRNA translocation (*k*_2_), and EF2•GDP release (*k*_3_). Reactions associated with ADPR•EF2 were depicted in gray, and the corresponding reaction rate constants distinguished by an asterisk superscript. (*B*) Model variations in the PRE ribosomal binding step and subsequent ADPR•EF2 turnover. The reversible factor binding and subsequent GTP hydrolysis are combined together as 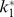 = *k*_a_ *k*_GTP/_(*k*_d_+*k*_GTP_). Three minor model variations are: 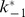 = 0 (model V1), 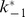 ≠ 0 and the dissociated factor is 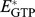 (model V2), and 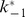 ≠ 0 and the dissociated factor is 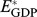 (model V3).

The model for the elongation cycle is depicted in Figure 1A with emphasis on the EF2-mediated events. Major assumptions are:

1. EF2 and another elongation factor, EF1α, bind alternatively to the same binding site on the ribosome during the cycle.
2. Ribosome-free EF2 always exists in a complex form with either GTP or GDP, denoted as *E*_GTP_ or *E*_GDP_, respectively, and 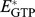 or 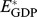 for their ADP-ribosylated counterparts.
3. In keeping with the disparate affinities of EF2/EF-G in different nucleotide-bound states to the PRE and POST ribosomes [14], [17], [28], the PRE ribosome binds with EF2 only in the GTP state, i.e., *E*_GTP_ or 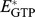.
4. The EF2-independent spontaneous translocation, reportedly infrequent and amounting to less than 1% of the activity catalyzed by EF2 [9], [10], is ignored.
5. All ribosomes are assumed to be polysomes.
6. Codon advancement by the tailing ribosomes along mRNA could be temporarily or permanently stalled, depending on the status of the preceding ribosome.
7. Only free EF2 is susceptible to, while the ribosome-bound EF2 is protected from, toxin modification [2], [13], [19], [29].
8. Toxins represent the catalytic fragments only; they ADP-ribosylate free EF2 in either GTP- or GDP-bound form indiscriminately and irreversibly (assuming negligible amount of nicotinamide).
9. NAD^+^ is in excess relative to EF2, making ADP-ribosylation an effective bimolecular reaction of EF2 and toxins.

The elongation cycle considers four sequential reactions: the EF1α-initiated polypeptide elongation (*k*_0_), factor binding followed by instant GTP hydrolysis (combined together as a one-way reaction by an apparent *k*_1_), the EF2-mediated tRNA translocation (*k*_2_), and release of deacylated-tRNA and EF2•GDP from the POST ribosome (*k*_3_). The phases of ribosomes interposed between the four reactions are the POST ribosome *R*_O_ (phase 0, poised to accept a cognate aminoacyl-tRNA in the vacant A site, indicated by the subscript “_O_”), the empty PRE ribosome 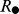 (phase 1, bearing deacylated tRNA in the P site and peptidyl-tRNA in the A site), the EF2-bound PRE complex 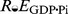 before full translocation (phase 2, with 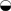 denoting the half-empty A site), and the POST complex *R*_O_ *E*_GDP_ (phase 3) after full translocation of the mRNA-tRNA_2_ duplex. Because GTP is hydrolyzed rapidly upon binding of *E*_GTP_ with 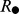 [9], we combine the reversible factor binding event and subsequent GTP hydrolysis into a unidirectional reaction characterized by an equivalent *k*_1_ for native EF2•GTP (Figure 1B), defined as *k*_1_ = *k*_a_ *k*_GTP_/(*k*_d_+*k*_GTP_). Here, *k*_a_ and *k*_d_ are respectively the association and dissociation rate constants of the reversible binding reaction, and *k*_GTP_ is the rate constant for GTP hydrolysis.

The hydrolyzed 80S•EF2•GDP•Pi complex 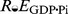 signifies the beginning of a tight coupling between the hydrolyzed factor EF2•GDP•Pi and the 80S PRE ribosome, during which significant conformational changes take place that eventually lead to translocation of the mRNA-tRNA_2_ duplex in the ribosomal intersubunit tunnel. Binding with EF2 stabilizes a ratchet-like intersubunit rotation and induces the ribosomal 40S head to swivel relative to the body [5]–[7]. These structural movements shift the 3′-CCA ends of the P- and A-site tRNAs in the large 60S subunit towards the E and P site, respectively, while the anticodon ends of both tRNAs remain anchored in the small 40S subunit, forming the hybrid positions. The precise mechanism in the following 40S translocation remains elusive except that it involves a series of further structural rearrangements within the complex [4]–[7]. For our purpose of studying the toxin-induced inhibition kinetics of protein synthesis, there is no need to plot an over-sophisticated translocation scheme. Consequently, we model translocation as an irreversible reaction by *k*_2_, which most likely reflects the slow unlocking process that precedes the 40S tRNA translocation.

After concerted translocation of the mRNA-tRNA_2_ duplex in the ribosomal 40S subunit, which is accompanied by sliding of one codon in the ribosome, the deacylated and peptidyl-tRNAs form the classic E/E and P/P sites, respectively, while the tip of domain IV of EF2 occupies the 40S A site to prevent tRNA back-movement [5]–[7]. The POST ribosomal complex *R*_O_ *E*_GDP_ undergoes further conformational reset, known as relocking or back-ratcheting, to release the deacylated tRNA and *E*_GDP_, thus returning the *R*_O_ phase again.

### ADP-ribosylation of Ribosome-Free EF2

The total rate of ADP-ribosylation of soluble EF2 is modeled as
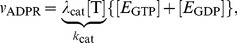
(1)wherein [T] denotes the toxin concentration and *λ*_cat_ is a bimolecular rate constant. Since toxin dose is not the focus of our investigation, we combine *λ*_cat_ and [T] together as a first-order *k*_cat_.

### Ribosome-ADPR•EF2 Interactions

The rate constants for the ribosomal interactions with ADPR•EF2 are distinguished by an asterisk superscript. Kinetic studies of prokaryotic translocation reveal that when tRNAs translocation is blocked, EF-G remains tightly bound within the PRE ribosomal complex. However, evidence also exits showing uncoupled Pi release/EF-G turnover from translocation [8–10, and references therein]. One relevant finding is that mutation of a highly conserved histidine (his583) or deletion of a few residues in domain IV of EF-G decreases the rate of translocation without affecting the turnover of mutant EF-G with the 70S ribosome [30]. To accommodate both possibilities, we append a “premature” turnover route 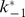 for 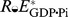 (Figure 1B) to allow dissociation of 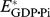 from the PRE complex 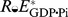. Two possibilities are considered. One (designated as model V1) assumes no premature turnover of ADPR•EF2 (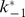 = 0), and another allows the premature turnover (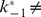 0) but at a much slower rate than the intact release rate *k*_3_. We further divide the finite 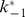 case into two scenarios according to the guanine nucleotide associated with the released factor (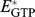 for model V2 and 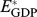 for model V3). The former is in line with an alternative model [31] advocating reversible GTP hydrolysis before translocation; the latter implies irreversible GTP hydrolysis regardless of the status of tRNA translocation. As for native EF2, we assume that its turnover takes place only after successful translocation via *k*_3_, meaning that the pathway *k*_−1_ does not exist for native EF2.

The elongation scheme is translated into a system of differential equations for the ribosomes in various phases:
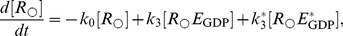
(2)




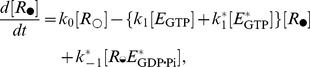
(3)

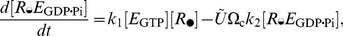
(4)

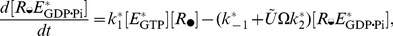
(4')

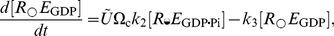
(5)

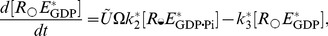
(5')wherein Ω and Ω_c_ are the translocation stall functions accounting for temporary and permanent translocation stalls, respectively. The drivations of Ω and Ω_c_ will be given shortly.

### Guanosine Nucleotide Exchange on Free EF2

The exchange of guanosine nucleotides on soluble EF2 is modeled as a reversible second-order reaction: 
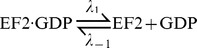


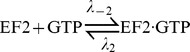



After substituting the quasi-steady-state for the free [EF2] into the rate equation for *E*_GTP_, 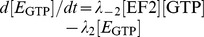, one obtains
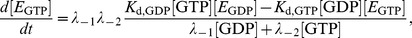
(6)where in *K*_d,GDP_ ( = *λ*_1/_ *λ*_−1_) and *K*_d,GTP_ ( = *λ*_2/_ *λ*_−2_) are the equilibrium dissociation constants as defined. We assume constant [GTP] and [GDP], setting them as [GTP] = 400 µM and [GDP] = 40 µM based on the approximate ratio [GTP] : [GDP] = 10∶ 1 in eukaryotes [32]. We further set *λ*_−1/_ *λ*_−2_ = 4 according to [32]. Upon substituting *λ*_−1_ = 4*λ*_−2_ and replacing the symbol *λ*_−2_ by *k*_4_, Eq. (6) simplifies to



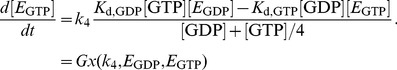
(7)If *K*_d,GDP_ and *K*_d,GTP_ are unaffected by ADP-ribosylation, the same functional form *Gx* holds for the rate of nucleotide changes in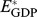 with a modified 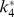.

### EF2 Cycle

EF2•GTP is cycled through the translocation step with the PRE ribosome and regenerated via the guanosine nucleotide exchange in the free phase. Since the diphthamide in domain IV is 60–70 Å away from the nucleotide-binding pocket in domain I of EF2 [25], we assume that toxins inactivate soluble EF2 associated with either nucleotide indiscriminately. The mass conservation equations for the GDP-bound EF2 are written as
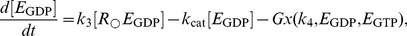
(8)and 
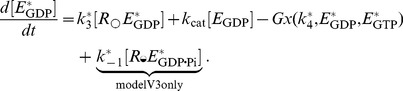
(8')


Similarly, the governing equations for GTP-bound EF2 are 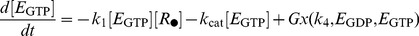
(9)and 
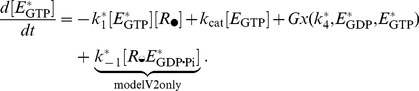
(9')


### Conservation of Amino Acids in the Cell-Free System

A constant *k*_0_ for the EF1α-mediated peptide elongation implies abundance of various amino acids and tRNAs during mRNA translation. When modeling the incorporation of radiolabeled amino acids or the poly(U)-directed phenylalanine synthesis, we assume that *k*_0_ could decline if some specific amino acid, such as ^14^C-phenylalanine (^14^C-Phe), is overly depleted. The rate of consumption of the free ^14^C-Phe, denoted as *C*_phe_, is correlated to its rate of incorporation by *dC*_Phe_/*dt* = -*v*. The dependence of *k*_0_ on *C*_phe_ is empirically set as
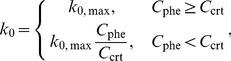
(10)in which *k*_0,max_ is the maximal value of *k*_0_ when ^14^C-Phe is ample, and *C*_crt_ is the concentration threshold (set to be 0.7 mM) for *C*_phe_, below which *k*_0_ starts to decline linearly.

### Translocation Stalling

Movement of the ribosome along mRNA may become temporarily stagnant if it happens to be in a *non*-translocating phase or follows behind a nontranslocating one. Because most of the eukaryotic ribosomes are assembled as polysomes, stagnant ribosomes may temporarily, or permanently in the case of ADPR•EF2, hinder the movement of other translating ribosomes, a prediction consistent with the increased polysomal fraction in eukaryotic lysates after toxin treatment [17], [33]. Consequently, translocation stalling due to non-synchronized or inhibited ribosomal activities must be considered.

Under the general assumption that ADPR•EF2 could still mediate translocation, we define the equivalent population fraction of the ribosomes in the translocation-active phase, *f*, as 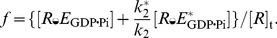
(11)


**Figure 2:**
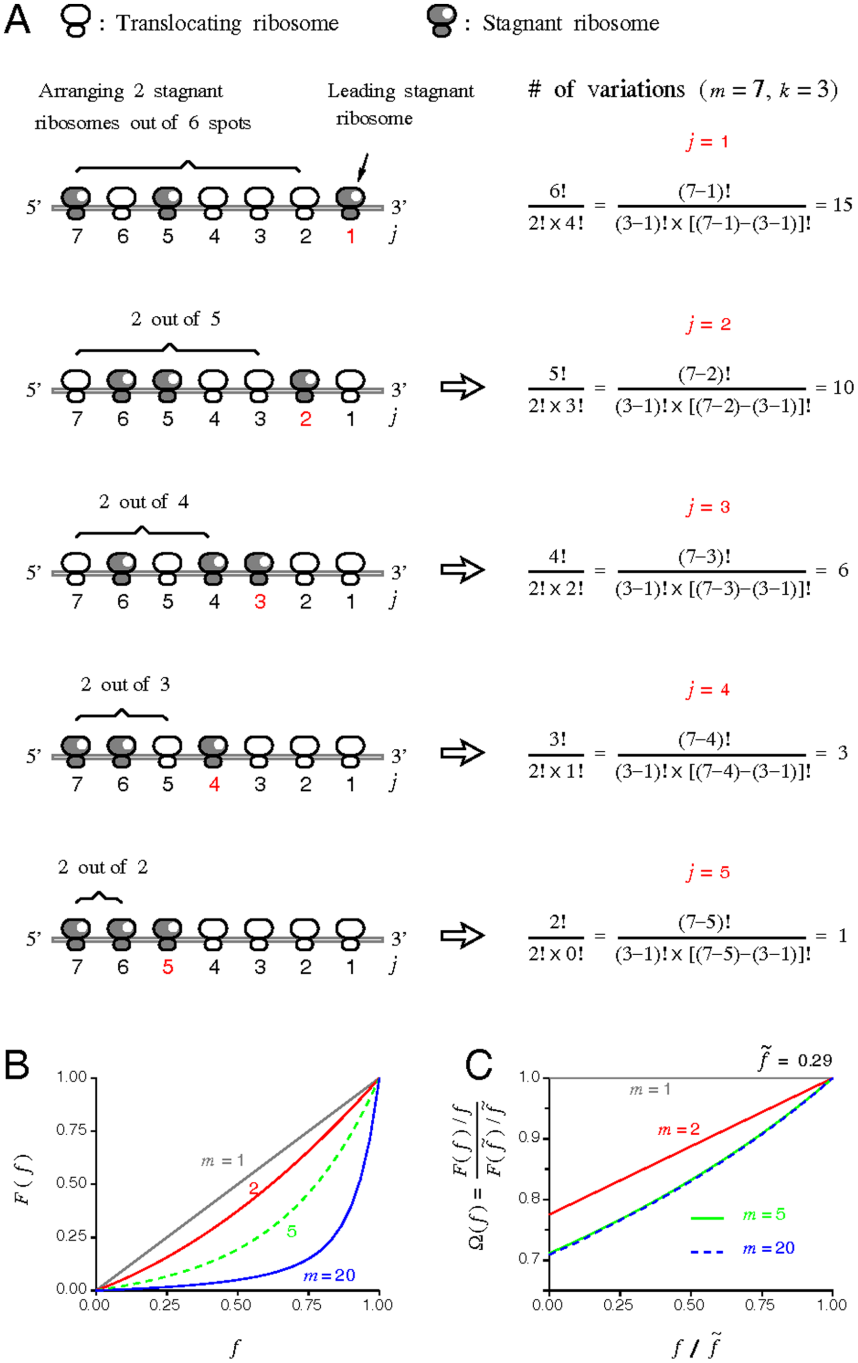
Statistical reasoning in constructing the temporary stall probability. (*A*) Cartoon illustration of arranging 3 stagnant ribosomes (*k* = 3) within an mRNA ORF containing 7 ribosomes (*m* = 7). Number of arrangement variations is given at the right column according to the position of the leading stagnant ribosome in the series, *j*. The formula is summarized as (*m*–*j*)!/{(*k*–1)![(*m*–*j*)–(*k*–1)]!}. (*B*) Plot of the actual translocating population *F* versus the translocation-active fraction *f*. (*C*) The stall function *U*(*f*) [ = *F*(*f*)/*f*] replotted in the normalized form 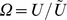 within a physiological range of *f*



Here, [*R*]_t_ is the ribosomal total concentration in a cell-free system or the cytosol of a single cell, and the ratio 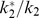 reflects the extent of translocation efficiency in 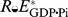 relative to the native group 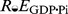. Assume that all open reading frames (ORFs) in mRNAs are of the same length and filled with the same polysomal density (*m* ribosomes per ORF). Using *m = *7 and *k* = 3 as an example, Figure 2A illustrates that the probability to find the *leading* stagnant ribosome at the *j*th position in the *m* series (counted from the 3′ end) follows the classic binomial distribution:

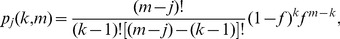
(12)which is valid for all *m* and *k* except for *k = *0. When *k = *0, the probability *p*_0_(0, *m*) is simply 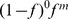. The use of *f* in Eq. (12) in the binomial probability expression implies a statistically uniform distribution of the translocation-active ribosomes on mRNA, a crude simplification.

We further assume that codon movement of all ribosomes trailing behind the leading *stagnant* one would be instantly affected. Hence, only the translocation-active ribosomes preceding the leading stagnant one get to advance their codon positions at that particular moment. The statistically averaged fraction of the actual translocating polysomes, *F*, is defined as multiplying the individual probability *p_j_*(*k*,*m*) to the number of the actual translocating ribosomes, *j*–1, followed by summing over *j* and *k* and, lastly, divided by *m*,
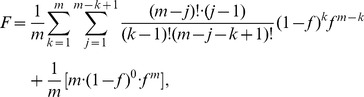
(13)wherein the last term represents the contribution from *k* = 0. By algebraic manipulations, Eq. (13) can be simplified to



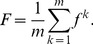
(14)Because of stalling, the fraction of actually translocating polysomes *F* is always less than the fraction of the translocation-active population *f* (Figure 2B), excluding the two end points at *f* = 0 (none translocated) and *f* = 1 (all translocated). Utilizing the definition of *f*, the true rate of ribosomal translocation is rewritten as 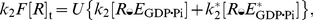
(15)in which *U*(*f*) [ = *F*(*f*)*/f*], defined as the *temporary* stall function, can be viewed as a population ratio of the translocated population *F* to the translocation-active population *f*. We can further express *U*(*f*) as



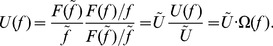
(16)Here, Ω is the normalized form of *U* and
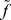
is the basal value of *f* in the absence of toxins. The advantage of expressing *U* in terms of Ω is that the normalized Ω becomes almost independent of the polysomal density *m* when *m* is larger than 5 (Figure 2C).

The actual extent of stalling could be more serious than being described by Ω(*f*) because of the coarse assumption for a uniform distribution of the translocation-active ribosomes on ORF. It is especially so if the ADPR•EF2-bound ribosome is unable to move along the mRNA (i.e., 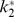 = 0). Note that unlike *F*, the normalized function Ω does not decline to zero at *f = *0 (Figure 2C). To account for permanent stall on the intact ribosomes induced by nontranslocating 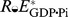, we introduce an empirical form of Ω_c_ for the intact 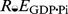, so that the total rate of translocation is the sum of 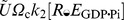 and 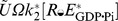. Arbitrarily, Ω_c_ is set identical to Ω unless the ADPR•EF2-bound 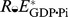 population exceeds 4% of [*R*]_t_ and the translocation-active fraction *f* declines to 9% of [*R*]_t_: 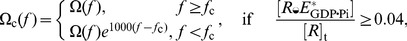
(17)in which *f*_c_ ( = 0.09) denotes a crtical level of *f* associated with a dead-end translocation block (i.e., Ω_c_ ≈ 0). Both conditions (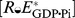 ≥4% of [*R*]_t_ and *f* ≤9% of [*R*]_t_) are set according to experimental findings in [17]. This *f*-capped Ω_c_ is associated with 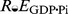 to ensure that some intact EF2 will eventually be frozen within the 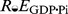 population inaccessible to toxins.

### Rate Expressions for Peptide Elongation and ADP-ribosylation

The rate of protein synthesis is conventionally measured as the rate of incorporation of radiolabeled amino acids into translated polypeptide chains. The apparent rate of protein synthesis is hence defined as the ongoing rate of the elongation cycle, 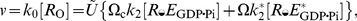
(18)assuming no differences in the incorporation of individual amino acids. Since the ribosome-bound EF2 is protected against toxin inactivation, ADP-ribosylation activity exists only in the soluble phase and can be described as *k*_cat_ ([*E*_GTP_]+[*E*_GDP_]).

### Numerical Procedures

The system of the twelve coupled ODEs in Eqs. (2)–(9′) is solved numerically by the fourth-order Runge-Kutta method adopting an adjustable time step with a minimum Δ*t* = 10 ms. An executable file and its numerical codes, written in Fortran90, are provided as Materials S1. Most simulations are performed on a model system made up of [EF2]_t_ = 0.6 µM and [*R*]_t_ = 0.5 µM. The value for the ADP-ribosylation rate constant *k*_cat_ = 0.0045 s^−1^ is equivalent to [T] = 0.16 nM or 250 toxin molecules in the cytosol of a cell. All other parameter values are listed in Table 1. The rate constants associated with an asterisk superscript are set identical to their native counterparts unless otherwise noted.

**Table 1:** Model parameters used in simulations.

	Symbol	value	unit	Comment
First-order rate constant for ADP-ribosylation	*k*_cat_	0.0045	s^−1^	assumed^1^
Total EF2 in the system	[EF2]_t_	0.6	µM/cell	[36]
Total ribosomes engaging in mRNA translation	[*R*]_t_	0.5	µM/cell	[37] 2
EF1-initiated peptidyl insertion rate constant	*k*_0,max_	4	s^−1^	[38]
Association rate constant between *E*_GTP_ and *R*_•_	*k*_1_	96	µM^−1 ^s^−1^	[8], [9] 3
Dissociation rate constant for 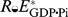	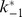	0.3	s^−1^	assumed
Translocation rate constant for 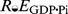	*k*_2_	35	s^−1^	[8], [9]
EF2•GDP release rate constant for 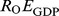	*k*_3_	5	s^−1^	[8], [9] 4
GTP exchange rate constant for *E*_GDP_	*k*_4_	12.8	µM s^−1^	[39]
EF2•GDP dissociation constant	*K*_d,GDP_	0.5	µM	[24]
EF2•GTP dissociation constant	*K*_d,GTP_	2.68	µM	[24]
Free GTP concentration	[GTP]	400	µM	[35]
Free GDP concentration	[GDP]	40	µM	assumed^5^
Polysomal density	*m*	10	# ORF^−1^	assumed
Threshold for dead-end translocation block in *f*	*f*_c_	0.09		[17]
Basal fraction of translocation-active ribosomes	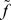	0.29		modeled
Basal stalling coefficient	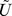	0.14		modeled

The rate constant 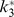 is set equal to *k*_3_ throughout the work, whereas 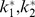, and 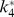 may differ from their native counterparts.

1 Calculated from *λ*_cat_ [T], using *λ*_cat_ = 1.7×10^9^ M^−1^ min^−1^ [34] and [T] = 0.16 nM (equivalent to 250 toxin molecules in the cytosol of a cell, with a cell cytosol volume equal to 2.6 pL [35].

2 Obtained by the ratio of [EF2]_t_ : [*R*]_t_ = 1.2∶ 1 [37] using the estimated [EF2]_t_.

3 Calculated as *k*_1_ = *k*_a_ *k*_GTP_/(*k*_d_+*k*_GTP_) = 150*250/(140+250) = 96 s^−1^.

4 Taken as the rate constant for relocking [8], [9].

5 Based on [GTP]:[GDP] = 10∶1 [32].

## Results

### Baseline Simulations (without Toxins)

Because kinetic data for the individual steps in the eukaryotic elongation cycle are mostly lacking, prokaryotic data are substituted whenever possible. These kinetic parameters, taken from prokaryotic systems without modifications, dictate that the EF1α-initiated chain elongation (*k*_0_ = 4 s^−1^) sets the pace of the cycle as a whole. Accordingly, most of the ribosomes in the cycle accumulate at the POST phase *R*_O_. The overall translation rate is slightly slower than typically found for eukaryotes (6∼10 codons per ribosome per second), but imposes no changes to our conclusions. The simulated percentage of the ribosomes in the phase *R*_O_ (35%) compares well with the reported puromycin-reactive polysomal population in rabbit reticulocyte (40%, [40]). The second slowest step occurs at the EF2-mediated tRNA translocation (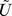 *k*_2_ = 4.85 s^−1^, *k*_2_ taken from [8], [9]), followed by the POST release of EF2•GDP (*k*_3_ = 5 s^−1^). The simulated value for the basal stall coefficient 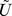 ( = 0.14, Table 1) implies that statistically, only ∼14% of the 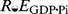 population (0.143 µM) undergoes codon advancement.

### Simulations with Toxins

#### Partial impairment

**Figure 3:**
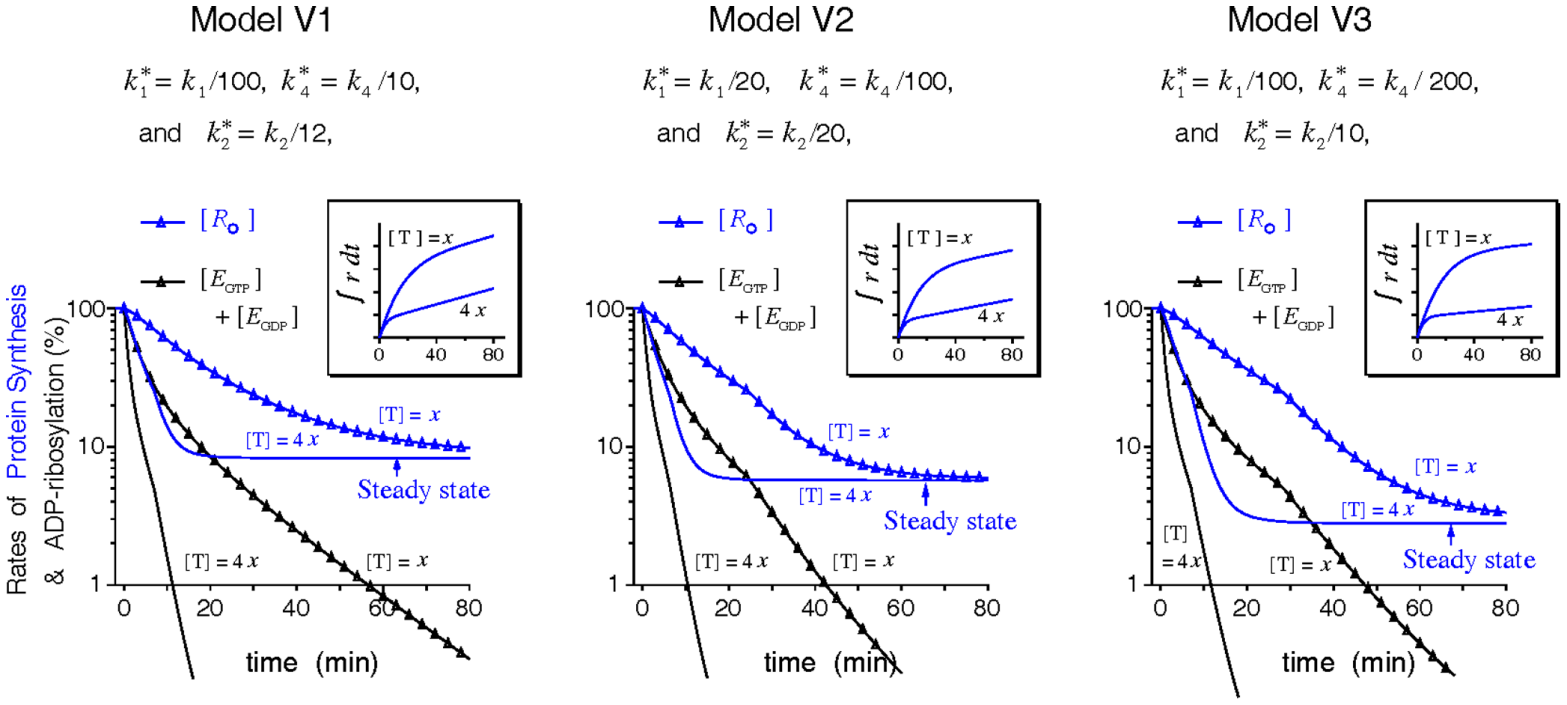
Effects of partial impairment in the ADPR•EF2-associated processes on the polypeptide elongation activities. The constant toxin dose *x* is set equal to 0.16 nM (equivalent to *k*_cat_ = 0.0045 s^−1^). Insets display the cumulative incorporations of amino acids over time.

The consequence of ADPR•EF2 impairment on any elongation process is represented by a reduction in the corresponding rate constant. Past works observed that the toxin-induced protein synthesis inhibition in cell lysates follows a log-linear decay [2], [3], [33]. However, Figure 3 demonstrates that partially impairing any of the elongation reactions associated with ADPR•EF2 simply lowers the overall rate of the elongation cycle to a new steady state, supported by the alternative pathway of ADPR•EF2. Changing toxin dose [T] does not alter the new steady rate but how fast the new steady rate is reached. This toxin dose-independent protein synthesis activity disagrees with past findings [3], [33]. The residual elongation activity sustains a steady rise of amino-acid incorporations over time (Figure 3 inset), which also contradicts most data showing a flat-line activity of amino acid incorporation following toxin intoxication. We therefore conclude that to produce an ever-lasting inhibition in the rate of protein synthesis, at least one of the three investigated parameters (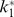, 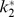, and 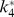) must be set zero, which gives rise to 2^3^–1 = 7 different *extreme* modes of ADPR•EF2 inhibition as defined in Table 2.

**Table 2:** Seven extreme modes (A to G) of ADPR•EF2 inhibition.

Modes	A	B	C	D	E	F	G
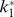				×	×	×	×
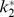	×	×			×		×
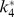		×	×			×	×

Rate constants 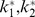, and 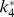 are assumed identical to their native counterparts, unless marked by the “×” letter, in which case the corresponding rate constant is zero.

#### Behaviors of different inhibition modes

**Figure 4:**
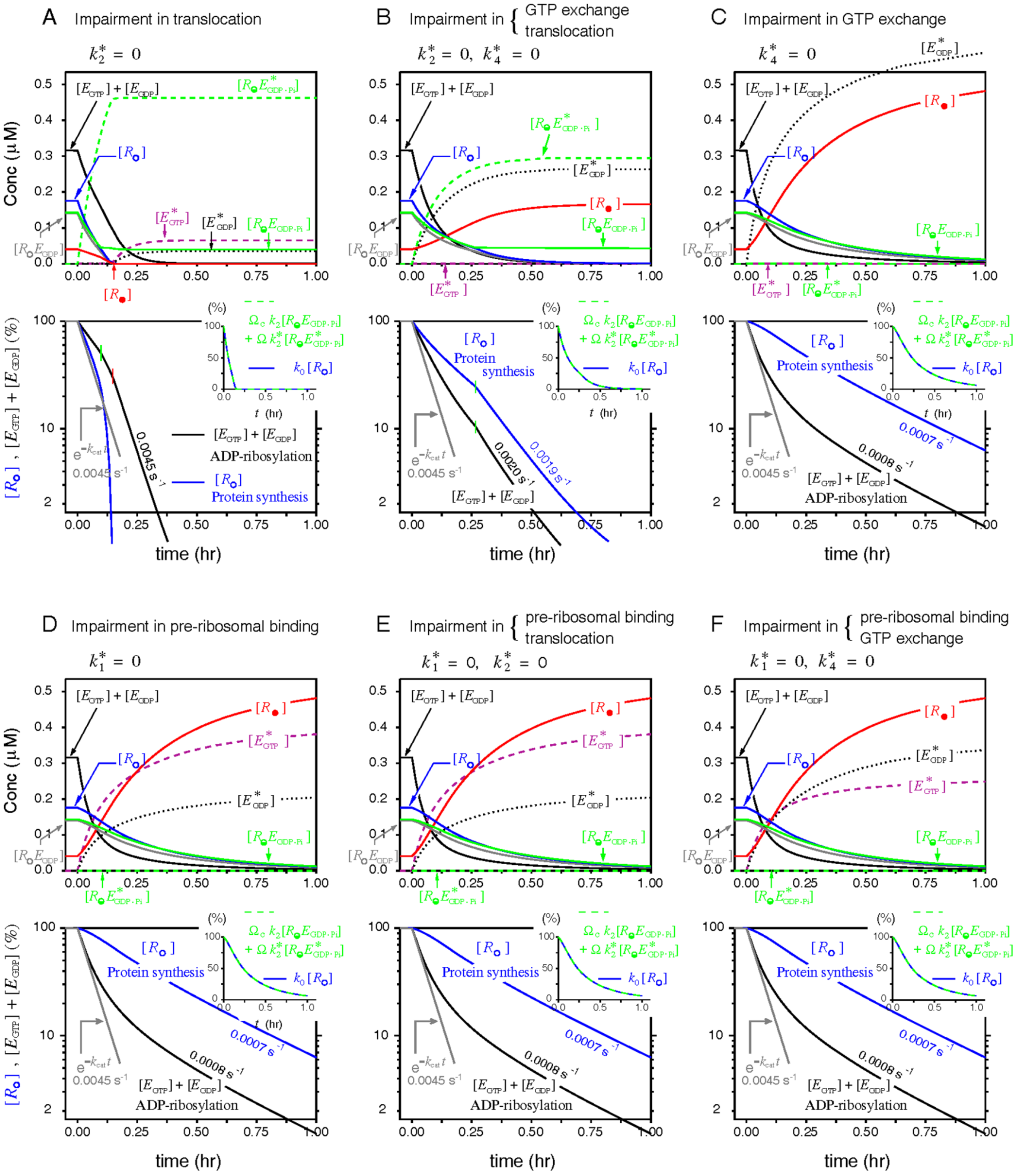
Transient ribosomal phase distributions and the decays of protein synthesis simulated by model V1 under the action of a constant toxin dose. Simulations were conducted using [T] = 0.16 nM (or *k*_cat_ = 0.0045 s^−1^) and the parameter values listed in Table 1 for the six inhibition modes defined in Table 2, with the mode G omitted. The normalized rates of the elongation cycle ([*R*_O_], blue lines) and of the inactivation of EF2 ([*E*_GTP_]+[*E*_GDP_], black lines) are drawn in semi-log scale. Nearby numerical values denote the apparent first-order inhibition slopes. A sharp transition of the inhibition slope is marked by a colored vertical line resulting from either a zero 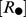 (red) or induction of the dead-end translocation block (green). Insets display superposition of the two normalized rate expressions Ω_c_ *k*_2_ 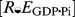
+ Ω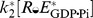 and *k*_0_[*R*_O_].

The phase distributions of the ribosomes and the log-linear decays of the rate of protein synthesis in the presence of a constant [T], as modeled by model V1 under various inhibition modes, are depicted in Figure 4. Similar results from the other two models are given in Figures S1 and S2. Several common features are observed. First, on a semi-log scale the rate of protein synthesis (represented by normalized [*R*_O_]) displays a first-order inhibition in parallel to that of EF2 inactivation (represented by normalized [*E*_GTP_]+[*E*_GDP_]) within the range of interest. The slope of this apparent log-linear inhibition is attenuated to various degrees compared to the intrinsic *k*_cat_. Second, the two rate expressions, *k*_0_[*R*_O_] and Ω_c_ *k*_2_ 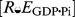 +Ω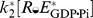, superimpose with each other well (Figure 4 inset), confirming that either expression could represent the percentage changes of the rate of protein synthesis. Third, the lagging correlation between EF2 inactivation and protein synthesis inhibition obtained from the inhibition mode A is opposite to that from other inhibition modes.

Note that in model V1, 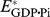 is not released from the complex 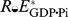 before translocation (i.e., 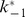 = 0). Hence, if the ADPR•EF2-mediated translocation is the sole inhibited process (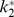 = 0, the inhibition mode A), 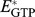 could bind and trap the PRE ribosome into an inactive and nondissociable state, leading to depletion of the functional ribosomes to bind with, and hence to protect, other free intact EF2. Once all PRE ribosomes are trapped by 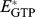 (signified by 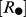 reaching zero in Figure 4A), the elongation cycle comes a complete stop. From this point forward, native EF2 is inactivated by toxins under no ribosomal protection, as reflected by the sudden increase of the log-linear inhibition slope to *k*_cat_, which is hence the ceiling level for the first-order ADP-ribosylation of EF2. In contrast, model V2 and V3 allow the ADPR•EF2-bound ribosomes to be slowly freed via the 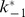 turnover route, thereby maintaining a finite 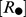 and slower inhibition kinetics under the same inhibition mode A. Whereas model V2 and V3 differ only in the nucleotide associated with the released factor, their simulation outcomes under the inhibition mode B are strikingly different.

One may also note the intact group 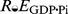 in the inhibition mode A or B being kept from further declines to zero and the sudden changes of the first-order inhibition slopes around *f* = *f*_c_. The former is attributed to the dead-end translocation block incurred by the accumulation of the nontranslocable 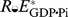 complex via the the zero-capped Ω_c_, for which 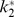 = 0 is the necessary but not the sufficient condition. The latter is associated with the explicit form of Ω_c_. Since Ω_c_ is arbitrary defined, we investigate the origin of the abrupt change in the first-order inhibition slopes in mode A and B, and how the explicit form of Ω_c_ affects the inhibition kinetics. This investigation is done by repeating the simulations in Figures 4, S1, and S2 with several different forms of Ω_c_ displayed in Figure S3. Using the inhibition mode B of model V1 as an exmaple, we show in Figure S4 that this abrupt change in the first-order inhibition slope originates from the tangential discontinuity of Ω_c_(*f*) when *f* is still higher than *f*_c_, not from Ω_c_ approaching zero (or *f* approaching *f*_c_). Though the explicit form in Ω_c_ does affect the apparent first-order inhibition slope slightly, this difference is insufficient to alter the main conclusions of this work concerning the pluasible modes of ADPR•EF2 impairment. As such, we continue to use the empirical Ω_c_ defined in Eq. (17) for the rest of modeling works.

#### Parameter sensitivity of the inhibition slope

The seven defined inhibition modes represent only the extreme cases. It can still be that one process is fully inhibited while the other two are only partially impaired. Fortunately, for most cases with one fully inhibited process, the apparent first-order inhibition slopes are insensitive to partial impairment of the other two, except when approaching their full inhibition (Figure S5). Another exception is the graded dependence of the apparent inhibition slope on 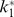 in model V2 and V3 when 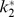 is inhibited, suggesting that 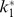 is a more critical parameter than the other two in influencing the overall inhibition kinetics of the system. Furthermore, different combinations of the parameters that give identical first-order inhibition slopes also yield identical inhibition profiles (Figure S6).

### Modeling of Experimental Data

#### Case 1

**Figure 5:**
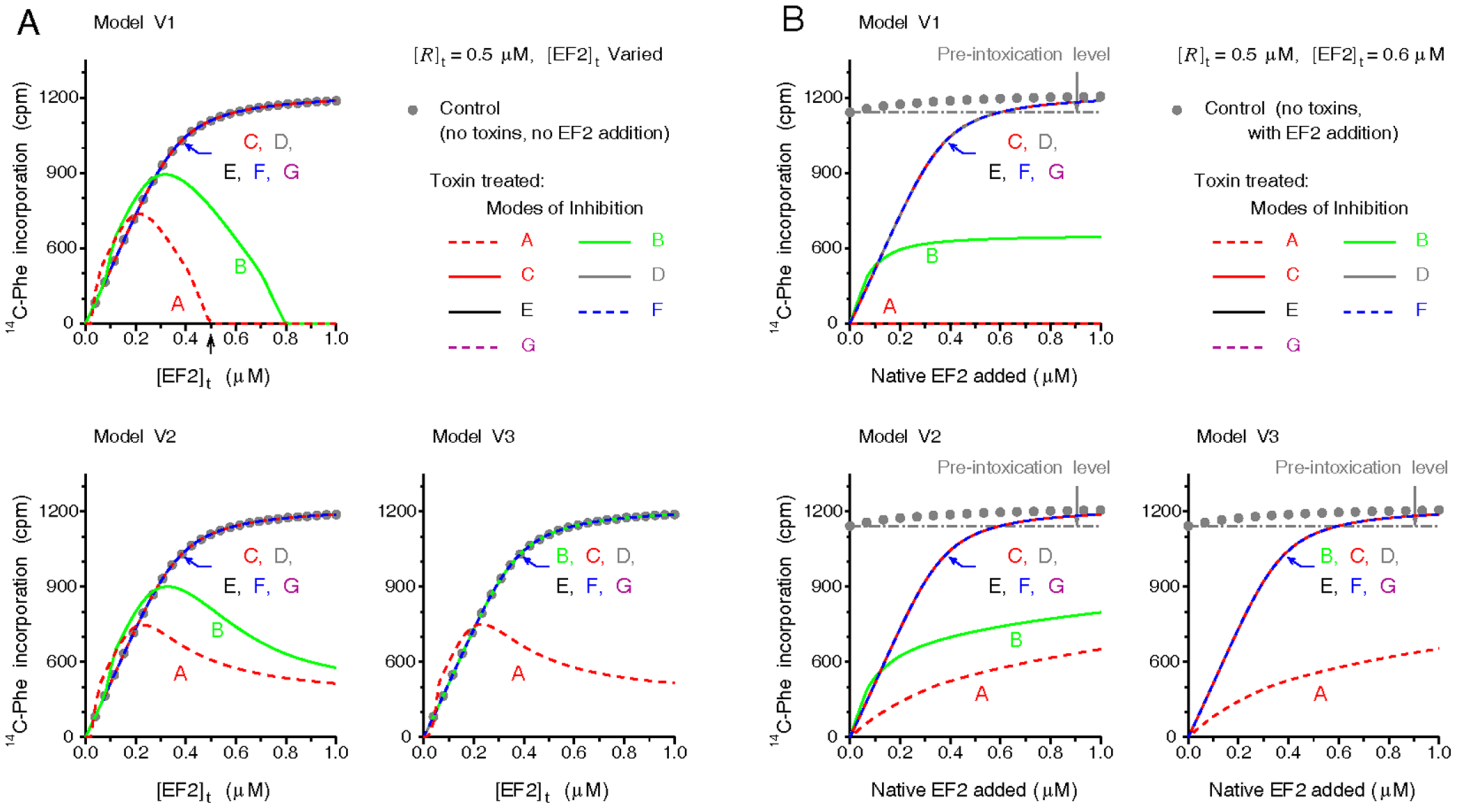
^14^C-Phe incorporation activities restored by additions of native EF2. (*A*) After thorough intoxication of a cell-free system made up of a constant [*R*]_t_ and varying [EF2]_t_, [T] was reset to zero and a specific amount of native EF2 equal to the system [EF2]_t_ and bolus ^14^C-Phe-tRNA in excess (6 mM) were added. Cumulative ^14^C-Phe incorporation for the next 40 min was calculated. Control dot (•) was obtained from a toxin-free system containing the same [*R*]_t_ and the [EF2]_t_ specified by the abscissa without the later EF2 addition. (*B*) Simulation procedures similar to (*A*), except that the system [EF2]_t_ was fixed to 0.6 µM. Namely, the added amount of native EF2 is independent of the original system [EF2]_t_. The control ^14^C-Phe incorporation (•) obtained under no toxins and no EF2 addition represents the pre-intoxication level of the system.

Prior works [1], [14], [29] observed that the amino acid incorporations from the lysates of intoxicated cells could be fully restored to their pre-intoxication levels, or even higher, by adding supernatants from normal cell extracts, gently washed ribosomes or purified EF2. The restoring power conferred by the washed ribosomes is attributed to the residual EF2 bound with the ribosomes [1], [29]. What inhibition modes would give similar restoration outcomes, however, remains unclear. To model the behaviors, we first allow a cell-free translating model system containing varying [EF2]_t_ to be intoxicated thoroughly by toxins for 2 hr. Then toxins are neutralized, fixed amounts of ^14^C-Phe and native EF2 equal to the original [EF2]_t_ are added into the intoxicated system, and the poly(U)-directed incorporations of ^14^C-Phe are counted for the next 40 min. As shown in Figure 5A, stoichiometric replacement of the intoxicated [EF2]_t_ in the system, wherein ADPR•EF2 acts by the inhibition mode A, elicits the least amount of ^14^C-Phe incorporations, followed by the inhibition mode B of model V1 and V2. This low restoration under the aforementioned inhibition modes is attributed to that most ribosomes in these modes are trapped by ADPR•EF2 by the time native EF2 is applied. Conversely, by the inhibition modes C to G, stoichiometric addition of native EF2 could restore the amino-acid incorporation in a thoroughly intoxicated system to exactly its pre-intoxication level. This is because ADPR•EF2 by these inhibition modes does not interact with the ribosome. Thus the inhibition modes C to G are more favorable over A and B when it comes to stoichiometric replacement of EF2. However, the usefulness of this conclusion is limited because in most relevant studies the system [EF2]_t_ was fixed, and EF2 was applied in excess to achieve the reported level of restoration. We next model in Figure 5B the ^14^C-Phe incorporation restored from an intoxicated system containing a fixed [EF2]_t_. In this case, the restored incorporation activity under the inhibition modes C-G (and B for model V3) turns out to be able to surpass the pre-intoxication level provided that sufficient native EF2 is applied (Figure 5B). This simulated behavior, qualitatively independent of parameter variations (Figure S7), is in agreement with the prior observations [1], [14], [29].

#### Case 2

**Figure 6:**
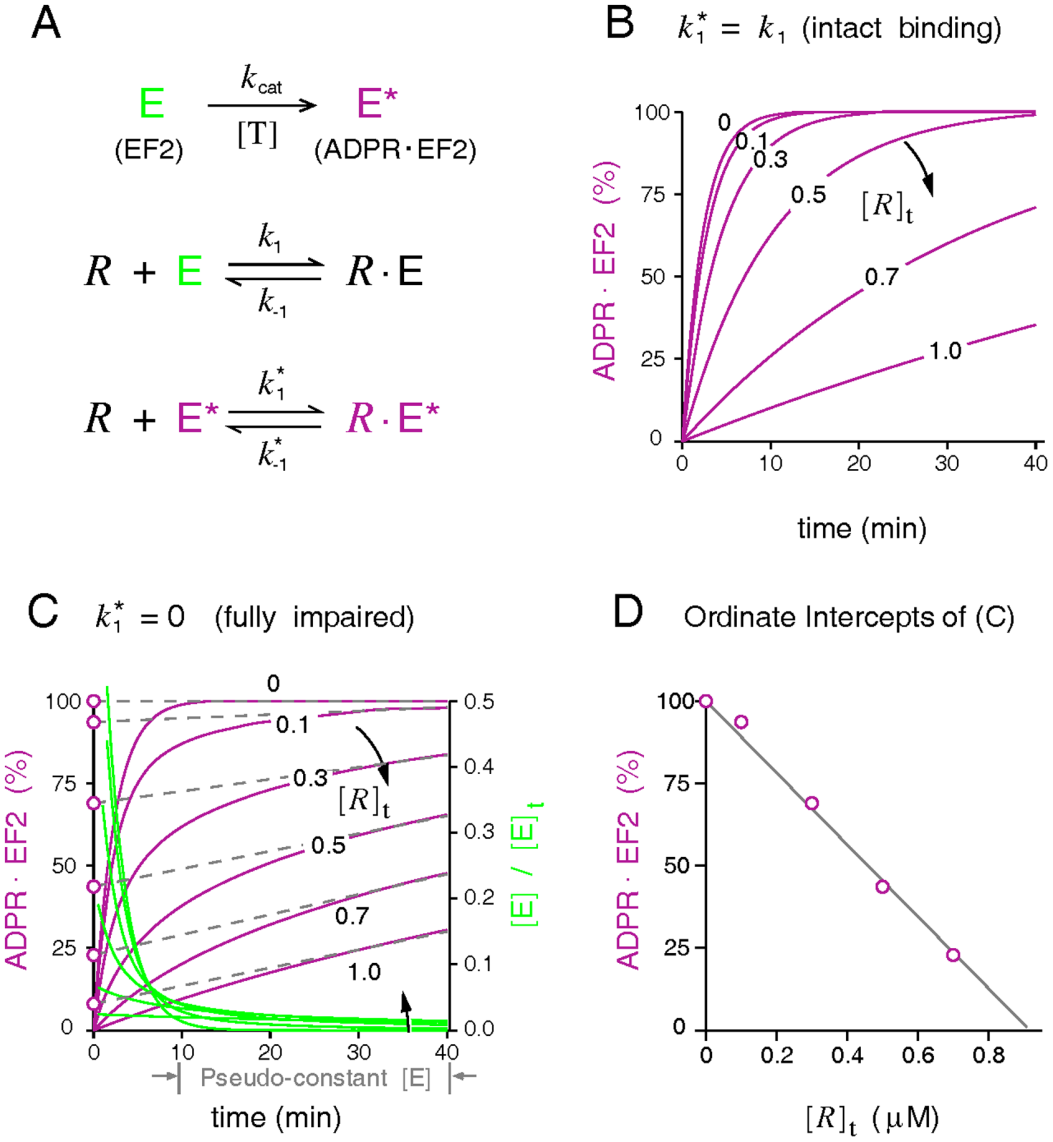
Protection exerted by empty ribosomes during ADP-ribosylation of native EF2. (*A*) The modeled reaction scheme, including the irreversible ADP-ribosylation of free EF2 (*k*_cat_) and two mutually exclusive reversible bindings of EF2 and ADPR•EF2 to the 80S empty ribosome. Simulations use the following parameters: *k*_1_ = 96 s^−1^ µM^−1^ and *k*_−1_ = 1 s^−1^ for native EF2, 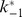 ( = *k*_−1_) for ADPR•EF2, *k*_cat_ = 0.0072 s^−1^, [EF2]_t_ = 0.6 µM, and [*R*]_t_ between zero and 1 µM. (*B*-*C*) Percentages of ADP-ribosylation, represented as ([E*]+[R•E*])/[EF2]_t_, simulated by an unaltered 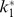 ( = *k*_1_) and a completely inhibited 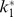 ( = 0), respectively. (*D*) Plot of the instant extent of ADP-ribosylation at time zero extrapolated from the pseudo-linear curves in (*C*) versus the system [*R*]_t_.

It is long recognized that the ribosome-bound EF2 is protected from toxin attack [2], [13], [19], [29]. To study the shielding effect of 80S empty ribosomes on EF2 reported in [2], we use a simplified scenario (Figure 6A), which portrays only the irreversible ADP-ribosylation of free EF2 and the reversible bindings of EF2/ADPR•EF2 with empty 80S ribosome. Modeling confirms the finding of [2] that the presence of empty ribosomes attenuates the rates, but not the eventual extent, of ADP-ribosylation. Furthermore, ADP-ribosylation in the presence of catalytic amounts of ribosomes goes to completion faster with an unaltered 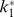 (ADPR•EF2 competes against EF2 for ribosomes, Figure 6B) than with an inhibited 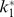 (ADPR•EF2 does not compete, Figure 6C).

Judging from the exhibited initial burst phase followed by a slowly rising phase that is quasi-linear with time, we conclude that the kinetic profiles of ADP-ribosylation depicted in Figure 6C are closer to those observed in [2]. Similar to the typical Briggs-Haldane enzyme systems, this quasi-linear phase of ADP-ribosylation results from the approximately constant level of the intermediate product, the free native EF2. The free native EF2 (green line, Figure 6C), following its initial rapid fall, attains a quasi-constant level established by an approximate mass balance between ADP-ribosylation and the dissociation of the ribosome-bound EF2. This quasi-constant level does not change substantially with [*R*]_t_. Hence if one draws a straight line tangential to the ADP-ribosylation curve at some time point in the quasi-linear region, the slope of the drawn straight line remains rather insensitive to [*R*]_t_. The ordinate intercept, extrapolated linearly from the quasi-linear ADP-ribosylation curves to time zero, has been interpreted in [2] as the percentage of free EF2 surplus to the ribosomes. Assuming that the initial burst phase of ADP-ribosylation is a result of rapid inactivation of these unbound factors by toxins, the authors of the aforementioned work [2] found that plotting the ordinate intercepts versus the system [*R*]_t_ yields an inverse linear relation in the catalytic range of [*R*]_t_; so do our extrapolations from the linear slope *k*_cat_[E] (with [E] estimated at *t* = 40 min) by the results of Figure 6C. Taken together, the simulations support the notion that ADPR•EF2 binding with 80S empty ribosome in [2] is impaired, thus delaying the ADP-ribosylation of free EF2.

#### Case 3

**Figure 7:**
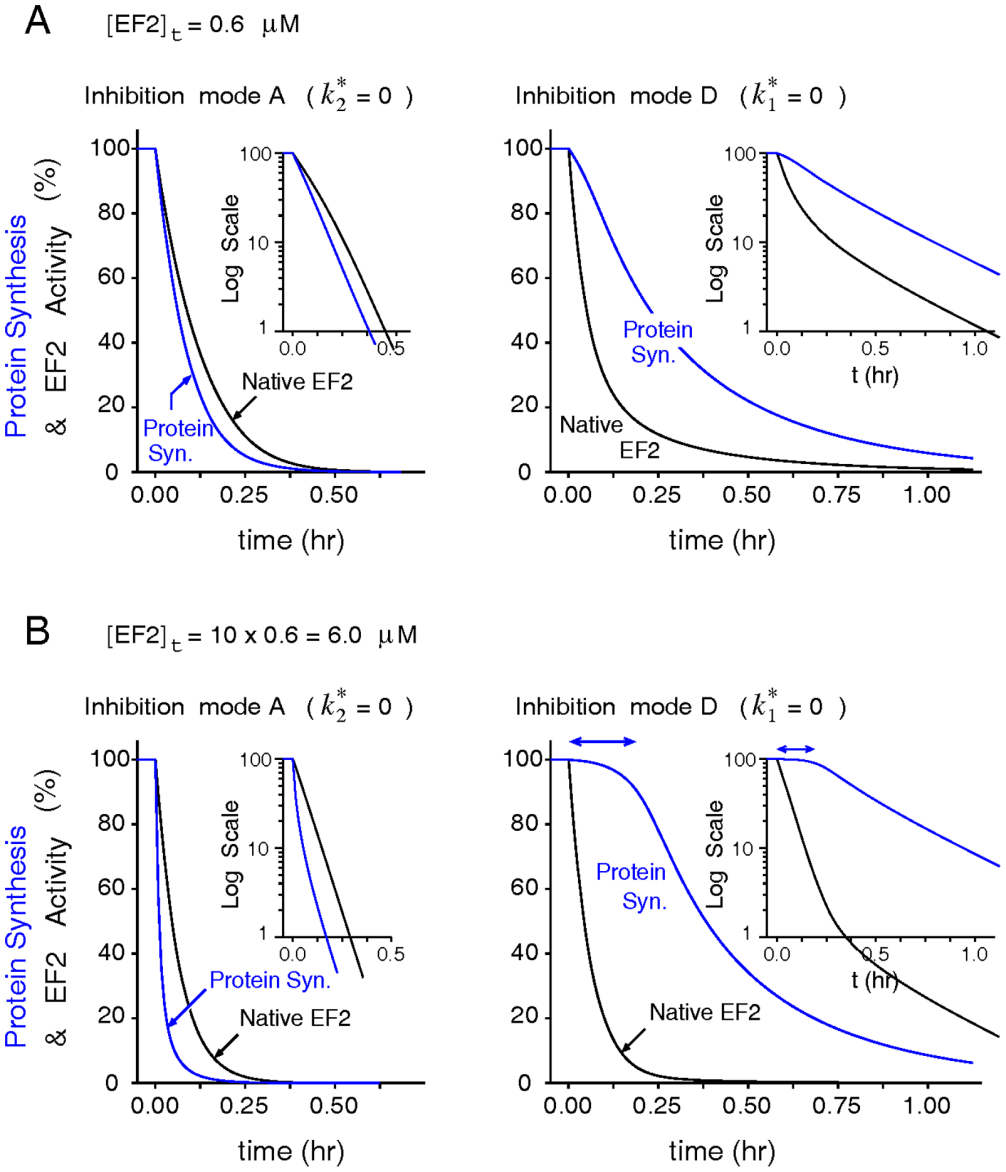
Delayed onset of protein synthesis inhibition by excessive EF2 could be produced only by modes inhibiting ADPR•EF2-ribosome interactions. (*A*) Comparisons of the transient declines in the protein synthesis rate and the intact EF2 concentration as modeled by the inhibition modes A (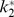 = 0) and mode D (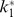 = 0) of model V3 (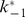 = 0.3 s^−1^) in a control system ([*R*]_t_ = 0.5 µM and [EF2]_t_ = 0.6 µM). Insets show the same profiles in semi-log-linear scale. (*B*) A tenfold increase in [EF2]_t_ lengthens the latency for the onset of protein synthesis decline in the mode D, but accelerates the inhibition of protein synthesis more than EF2 inactivation in the mode A.

Pappenheimer and colleagues [2], [41] once assayed the inactivation kinetics of protein synthesis and of intracellular EF2 in mammalian cells treated with diphtheria toxins, and found that the intracellular level of active EF2 declined rapidly once toxins reached the cytosol. In the meantime, the intoxicated cells showed no sign of decline in the rate of proteins synthesis for another hour. They attributed the delayed onset of protein synthesis inhibition to the time taken by the toxin molecules to inactivate most EF2 surpluses before inflicting a detectable damage on the ribosomal translating machinery. Figure 7 demonstrates that the onset of protein synthesis inhibition would lag behind EF2 inactivation only when the formation of the toxin-modified 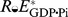 is abolished (e.g, 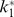 = 0), using the inhibition mode A and D under model V3 as an example. Increasing the system EF2 content further lengthens the latency for the decline of protein synthesis in mode D but inadvertently accelerates the inhibition of protein synthesis more than the inactivation of EF2 in mode A (Figure 7B). To assess if other inhibition modes consistent with mode D also produce a [EF2]_t_-prolonged latency in the intoxication of protein synthesis and the sensitivity of this behavior with model parameters, we calculated the changes of the half time *t*_1/2_ (the time at which protein synthesis declines to 50% of its initial value) with respect to 5% increases of [EF2]_t_ under a wide range of parameter variations (Figure S8). As shown, the gradient of the half time with respect to the system [EF2]_t_ is positive for all inhibition modes consistent with mode D but negative for the inhibition mode A of all models and the mode B of model V1 and V2. The mechanism behind this prolonged latency could also explain the “latent toxin dose” regime often formed in the toxin-dose response curve in?vitro [42] (Figure S9). Taken together, these simulation results hence discredit inhibition modes A and B as the underlying inhibitory mechanisms exerted by ADPR•EF2.

#### Case 4

The most straightforward approach to reveal the kinetic steps blocked by ADPR•EF2 during toxin intoxication is to examine the distributions of the ribosome, EF2, and ADPR•EF2 in each phase of the cycle. Nygård and Nilsson [17] had conducted such investigations. In their works, incubation of rabbit reticulocyte lysates with diphtheria toxins converted all unbound EF2 to ADPR•EF2 and reduced the fraction of factor-bound ribosomes to half (25% → 12%), over two-third of which remained associated with native EF2 (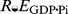 at 8.5%) while the rest with ADPR•EF2 (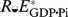 at 3.5%). This low level of 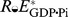 found in [17] is certainly not due to incomplete toxin intoxication since all free factors were ADP-ribosylated following prolonged treatment with excess nicked toxins.

**Figure 8:**
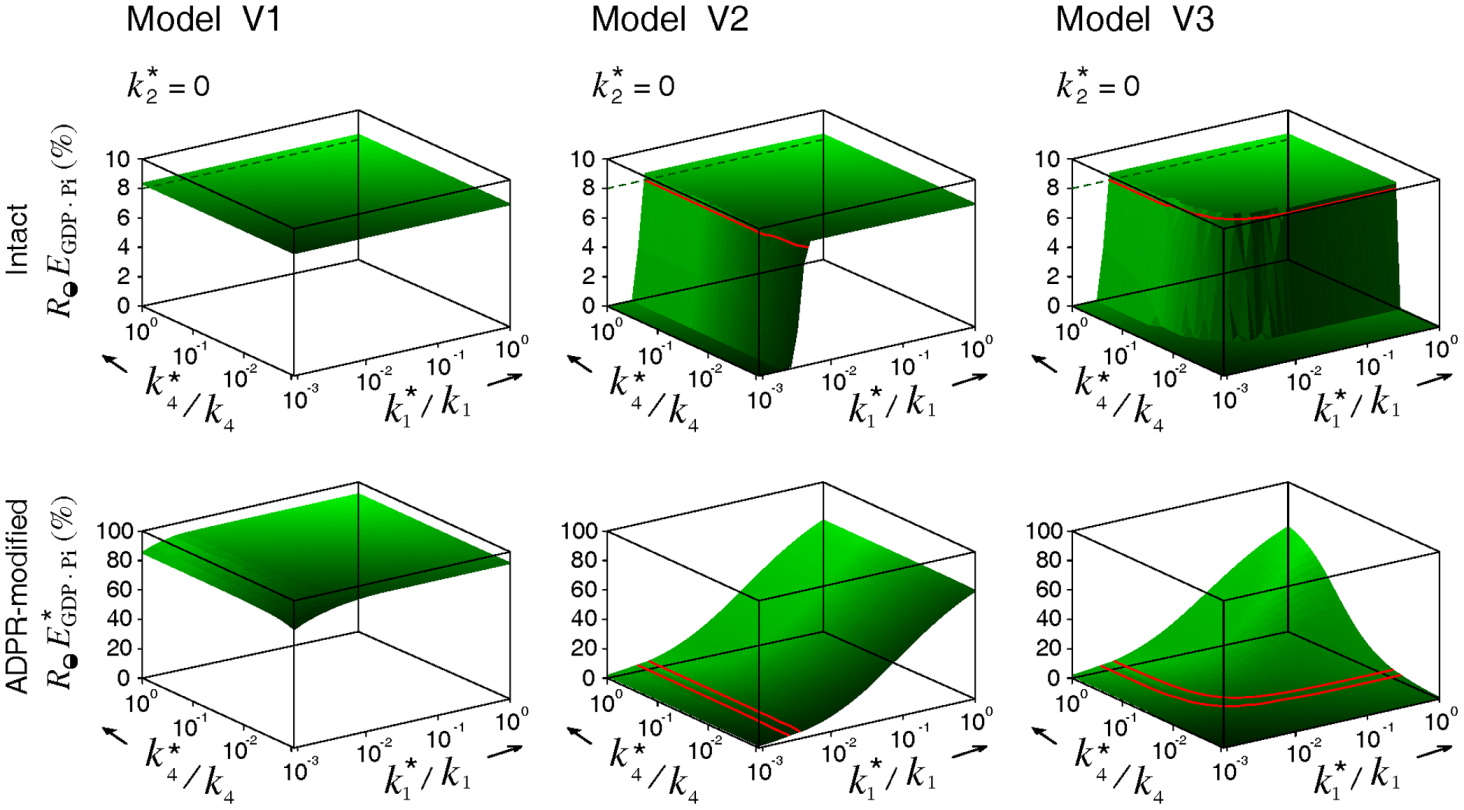
Optimal ADPR•EF2 parameters that produce simulation results consistent with Nygård and Nilsson [17]. In accordance to the experimental settings in [17], the final populations for the intact 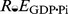 and the ADPR•EF2-modified 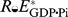 were obtained from a cell-free system made up of [EF2]_t_ = 1.1 µM (82 pmole) and [*R*]_t_ = 1.0 µM (75 pmole). This system was intoxicated by 1.2 µg toxins in 100 µL solution (equivalent to *k*_cat_ = 5.3 s^−1^) for 30 min. A higher premature turnover rate constant 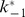 ( = 3 s^−1^) was used in the simulations of model V2 and V3. The red contour lines mark the levels for intact 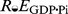 at 8% and ADPR-modified 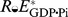 at 4%–5%.

We modeled the data of [17] to explore what combinations of the three parameters (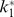, 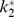 and 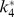) would produce final populations of 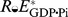 close to 4% and of 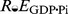 to 8.5%. The search for optimal parameters is simplified by the knowledge gained from our prior simulations (Figures 4, S1, and S2) that 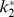 = 0 is a necessary condition to preserve the intact phase 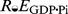. Figure 8 shows that while all three models could preserve the intact 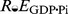 at 8%, the desired low 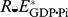 level (indicated by the green contour lines marking 4% and 5%) could result only from model V2 and V3 with a substantial reduction in either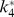 or 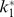 (or both), thus excluding model V1 as a potential candidate. Note that the simulations in Figure 8 are produced by a higher premature turnover rate 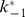 = 3 s^−1^ comparable to the normal turnover rate *k*_3_ = 4 s^−1^. A lower 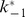 value only shifts the contour line of 4% 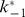 towards further reductions in 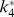 and 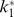, and vice versa (data not shown). Finaly, modeling of this case also stipulates that 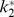 = 0 is a necessary condition to produce data-consistent simulations.

#### Case 5

**Figure 9:**
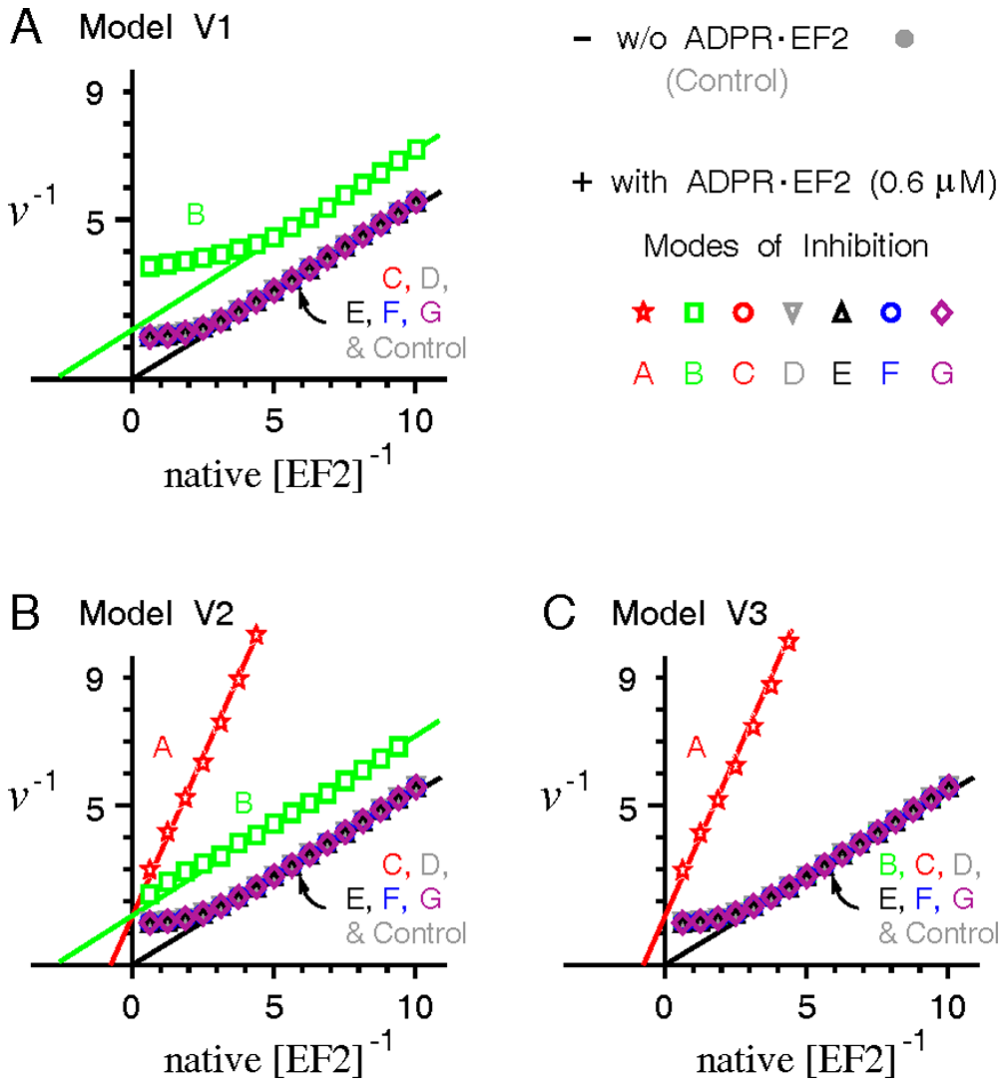
Double reciprocal plots of the elongation velocity *v* versus native EF2 in the absence and presence of a fixed amount of ADPR•EF2. A mixture of ADPR•EF2 (0.6 µM) and native EF2 in various concentrations was added into a cell-free poly(U)-translating system containing a constant [*R*]_t_ = 0.5 µM. The resulting stead-state elongation velocity *v* was recorded and plotted reciprocally versus the concentration of native EF2. Note that the elongation velocity *v* under the inhibition mode A of model V1 is zero and hence could not be displayed in the reciprocal form.

At last we present an opposite case favoring the inhibition mode A. In a work assessing the inhibitory effect of ADPR•EF2 in an artificial poly(U) translating system [23], ADPR•EF2 was found to increase the slope of the double reciprocal plots of the initial elongation velocity, while decreasing the negative intercept of the reciprocal EF2 (as substrate). Simulations of the double reciprocal plot of the elongation velocity *v* versus native EF2 (Figure 9) indicate that only the inhibition mode A of model V1 or V2 produces a steep slope compatible with the finding of [23]. By contrast, inhibition modes C-G prevent ADPR•EF2 to interact with the ribosome, leading to a less steep slope in the double reciprocal plot coinciding with the control (without ADPR•EF2). Similarly, sensitivity analyses (Figure S10) confirm that varying model parameters would not change the trend that the double reciprocal slopes in the mode A are steeper than in other inhibition modes. In terms of classic enzyme kinetic theories, ADPR•EF2 under the inhibition mode A exhibits a mixed-type binding competition to inhibit the ribosome-mediated protein synthesis, implying that ADPR•EF2 binds competitively against EF2 to inhibit the subsequent translocation step. ADPR•EF2 acts as uncompetitive inhibitor under the inhibition mode B (in model V1 and V2 only), and not an inhibitor at all under inhibition modes C to G. Because the elongation velocity *v* of model V1 under the inhibition mode A is essentially zero, no corresponding double reciprocal plot could be obtained. This inability to display the double reciprocal result for the inhibition mode A of model V1 consistently rejects model V1 as a credible model for describing the ADPR•EF2-ribosome interaction.

## Discussion

Despite the various modes of action ever suggested for ADPR•EF2 to inhibit protein synthesis, it remains unclear which one carries the decisive influence. As conflicting results and conclusions are common among studies, modeling may prove useful in analyzing the controversies.

### Analyses of Model Behaviors

In the current modeling study, the seven extreme inhibition modes yield varying levels of attenuation in the apparent first-order inhibition slope relative to the intrinsic *k*_cat_. Regardless of the model type, the greatest attenuation of the inhibition kinetics takes place when either 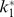 or 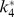, or both, is zero. This is because both parameters represent the upstream processes of the ADPR•EF2-mediated translocation event. Consequently, inhibiting either process maximally preserves the pool of functional ribosomes to interact with native EF2 in the intact elongation pathway. In that sense, the extent of attenuation in the inhibition kinetics reflects a protective capacity exerted by the ribosomes in the system to defer the toxin-catalyzed ADP-ribosylation of EF2, and hence the inhibition of protein synthesis. This protective capacity is diminished if ADPR•EF2 could trap ribosomes into translocation-inactive complexes, thereby reducing the pool of active ribosomes engaged in mRNA translation.

In summary, any mode of ADPR•EF2 inhibition or interactions with the ribosome that prevents the formation of the inactive 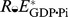 shall maintain a higher capacity in the system to defer the toxin-catalyzed inhibition of protein synthesis, and vice versa. This translocationally inactive complex would be less produced if the steps prior to the complex formation are blocked or if a premature turnover of ADPR•EF2•GDP, but not ADPR•EF2•GTP, take places actively. This conclusion is valid under the implicit assumption of one EF2 binding site per polysome.

### Data Comparisons

Simulations of partially impaired processes (Figure 3) suggest full inhibition of at least one process by ADPR•EF2, as confirmed by the pseudo-first-order inactivation of protein synthesis and native EF2 within the range of interests in Figures 4, S1 and S2. The simulation results of *Case 4* (Figure 8) further suggest the ADPR•EF2-mediated translocation to be the fully inhibited process (i.e., 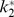 = 0). The restored protein synthesis activities modeled in *Case 1* (Figure 5) disfavor the inhibition modes A and B, consistent with the modeling results in *Case 3* for the delayed protein synthesis inhibition (Figure 7) and the latent toxin dose found in vitro (Figure S9). The kinetic profiles for ADP-ribosylation of EF2 in the presence of empty 80S ribosomes (Figure 6) similarly imply the inability of ADPR•EF2 to bind strongly with empty ribosomes. In *Case 4*, we observe that the phase distribution data of [17] could be reproduced only if model V2 or V3 depicts the correct ADPR•EF2–ribosome interactions and if either one of the ADPR•EF2-associated upstream events is severely impaired to limit the 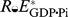 formation, despite the high turnover rate for 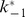. This observation rejects model V1, thereby favoring the possibility of a premature turnover of 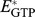 or 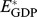 from the complex 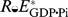. Moreover, we show that the conclusions based on the model simulations are robust to errors and deviations in the rate constants of the eukaryptic elongation cycle, and cannot be swayed by variations in the model parameters within the tested ranges.

By contrast, the strong binding competition exerted by ADPR•EF2 demonstrated in [23] supports a sole inhibition in the ADPR•EF2-mediated translocation (corresponding to the inhibition mode A) under model V2 or V3 (*Case 5*, Figure 9). Even so, simulations of this case also reject model V1 (i.e., no ADPR•EF2 turnover), which is consistent with other data. Interestingly, one prior study once hypothesized that ADPR•EF2 could support one round of peptide chain elongation before halting the translating ribosomes at the PRE state [12]. This hypothesis is compatible with a sole inhibition of 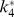 in the models (Figures 4*C*, S1*C*, and S2*C*), though more independent evidence is needed to validate this hypothesis. We recognize that some controversies in prior works are fundamentally irreconcilable, and more studies are needed to resolve the controversies. Therefore, it is impossible to render unifying conclusions or explanations at this moment. Taking all into considerations, we propose the following most plausible scenarios depicting the interactions between ADPR•EF2 and the ribosome. In addition to fully blocking tRNA translocation (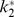 = 0), ADPR•EF2 exerts a substantial impairment on either of its upstream events described by 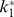 and 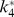, or both. The impairment on these upstream processes is too severe to be reconciled with the opposite contention that ADPR•EF2 could bind the PRE ribosome competitively. Though ADPR•EF2 could bind with the PRE-ribosome, the bound ADPR•EF2 dissociates rapidly from 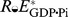 after the translocation step is inhibited (model V2 and/or V3).

The notion for a premature turnover of ADPR•EF2 is supported by prokaryotic studies showing that EF-G turnover is not necessarily coupled to tRNA translocation [30]. However, it remains uncertain whether the factor prematurely released from 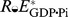 is associated with GTP or GDP. Though the conventional view is that GTP hydrolysis is irreversible, a recent study advocated a “reversible” hydrolysis of GTP before the tRNA P/E formation in the 70S•EF-G•GDP•Pi complex [31]. While this study may not necessarily be relevant to the eukaryotic case of toxin-inhibited tRNA translocation, more eukaryotic studies are certainly required to examine the possibility of futile GTP hydrolysis in the presence of ADP-ribosylating toxins or ADPR•EF2.

### Dead-End Translocation Block

One may argue that the occurrence of a permanemt translocation block on all intact EF2-ribosome complexes needs not be restricted to a low intact fraction *f*. While adopting a Ω_c_ that is capped to a higher *f*_c_ would bring the slope shift to an earlier time and defintely change the details of the inhibition profiles (Figures S3 and S4), it would not change the relative behaviors between different modes of ADPR•EF2 inhibition and hence our main conclusions in this work. Interestingly, the fraction of the ribosomes bound to intact EF2 assayed in [17] is rather low, corresponding to *f* = 8% at the end of toxin intoxication. Other independent work [29] has also found residual traces of native EF2 bound to ribosomes in thoroughly intoxicated cell extracts. In theory, the dead-end translocation block could occur whenever one single non-translocating 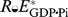 complex is formed. Plausibly, the reason that translocation block does not occur immeditaely is due to the replacement of the ribosome-bound 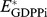 in 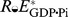 by an active *E*_GTP_, thereby resuming the suspended translocation event. However, the *E*_GTP_ replacement is possible only if (i) the ribosome-bound 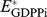 could dissociate from 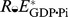 first and (ii) the soluble phase still contains native EF2, which is usually so in the beginning of toxin intoxication. Alternatively, the non-translocating 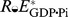 might dissociate itself from the mRNA to remove the block. This possibility is unlikely since toxin intoxication did not accelerate the breakdown of polysomes to single ribosomes [1] but increased polysomal fraction [17], [18].

### Relevant Structural Analyses

Structural studies already reveal that domain IV of EF2 shares intimate interactions with helices 34 and 44 of the 18S rRNA (part of the decoding center) and with the codon-anticodon stem loop of the P-site tRNA [6], [7], [43]. Current view is that before or during the full translocation event, the tip of domain IV of EF2 must sever the connection between the mRNA-tRNA duplex and the ribosomal decoding center [5], [7] while stabilizing the codon-anticodon base pairing at the same time [6], [25]. Consequently, it is conceivable that attaching a bulky ADP-ribosyl moiety to the tip of domain IV, wherein the diphthamide resides, may sterically impede the potential interactions of domain IV with its molecular partners, such as the P-site bound tRNA [6], [43] or the flipped-out A1492 and A1493 in helix 44 [44]. This speculation is in line with our conclusion for an inhibited 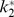. Surprisingly, no obvious conformational differences were detected from the cryo-electron microscopy (EM) reconstructions of yeast EF2 and ADPR•EF2, when in complex form with P site-charged 80S ribosome and a nonhydrolyzable GTP analog GDPNP, except for the ADPR density mass protruded from the tip of domain IV of ADPR•EF2 [7].

Perhaps the most controversial issue among relevant literatures is whether ADPR•EF2 in the GTP state could bind competitively with the PRE ribosome and induce GTP hydrolysis. Simulations in *Case 4* based on the data in [17] predict a seriously impaired PRE ribosomal binding/GTP hydrolysis event at 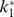
∼ *k*_1_/300 (Figure 8). This prediction agrees with [14] reporting decreased ribosomal affinity of ADPR•EF2 by two orders of magnitude and reduced GTPase activity by 50%, but disagrees with others [11], [23], [25]. Plausibly, the decreased rRNA binding may be explained by the repulsive charge interaction between the negatively charged ADP-ribosyl group and the rRNA phosphate backbone. In support, it had been shown that ADP-ribosylation decreased the binding affinities of EF2 with nonspecific RNAs [15] and with synthetic oligoribonucleotides mimicking the sarcin-ricin loop of the 28S rRNA [16]. Charging the P site with a tetrapeptidyl-tRNA [4], [28] or mutating a key nucleotide in the 23S rRNA of E. coli ribosomes [31] to block tRNA translocation was similarly accompanied by reduced apparent affinities of EF-G·GDPNP and EF-G·GTP to the PRE ribosome. Though stable bindings of ADPR•EF2 with either empty or charged 80S ribosomes had been demonstrated [7], [11], [25], we note that this demonstration was made only in the presence of nonhydrolyzable GTP analogs, which were known to stall EF2/EF-G turnover from the bound ribosome [9], [10], [25]. Thus the observed strong binding between ADPR•EF2 and the PRE ribosome may be due to the act of nonhydrolyzable GTP analogs freezing the ADPR•EF2-ribosome complex. Furthermore, the fact that toxins in the presence of nicotinamide could catalyze the reverse reaction of ADP-ribosylation [33], [41] also supports the idea of reduced affinity of ADPR•EF2 to the ribosome: toxins cannot catalyze the reverse reaction if the tip of domain IV of ADPR•EF2 is buried deep in the ribosome. In summary, we conclude that toxins inhibit protein synthesis mainly by depleting native EF2 to suspend the EF2-catalyzed translocation, instead of converting EF2 into ADPR•EF2 that actively traps and disables the translating ribosome.

## Supporting Information

Figure S1
**Transient ribosomal phase distributions and inhibition of protein synthesis from model V2 under simulation conditions identical to** **Figure 4** **.** The premature turnover rate constant 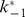 is set as 0.3 s^−1^.(TIFF)

Figure S2
**Transient ribosomal phase distributions and inhibition of protein synthesis from model V3 under simulation conditions identical to** **Figure 4** **.** The premature turnover rate constant 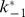 is set as 0.3 s^−1^.(TIFF)

Figure S3 **Arbitrary definitions of the permanent stall function Ω_c_.** Assume that Ω_c_ is expressed as the multiplication of *z*(*f*) and Ω(*f*), wherein *z*(*f*) is a unit function of *f*. Six different forms of *z*(*f*) are arbitrarily assigned in (*A*-*F*), with the common feature that they all monotonically decrease to near zero as *f* approaches *f*_c_ ( = 0.09). The tangential discontinuity in *z*(*f*) at the *f*_c_ upstream location is marked by a dashed circle.
(TIFF)

Figure S4 **Simulation results under the inhibition mode B of model V1 using the six empirical Ω_c_ defined in Figure S3.** Simulation conditions are identical to Figure 4. It is found that the sharp slope change in the inhibition profiles of protein synthesis and native EF2 results from the tangential discontinuity of Ω_c_ at the *f*_c_ upstream location, not from Ω_c_ approaching zero at *f*_c_.
(TIFF)

Figure S5
**Dependence of the apparent first-order inhibition slope on partial impairment of the ADPR•EF2 parameters.** Simulation conditions are identical to Figures S1 and S2 except for the settings of 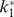, 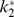, and 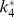.(TIFF)

Figure S6 **Identical apparent inhibition slopes often yield the same kinetic profiles in the inhibition of protein synthesis and ADP-ribosylation of native EF2.** Selection of the ADPR•EF2-related rate constant parameters is based on the simulated slope profiles in Figure S5.
(TIFF)

Figure S7
**Parameter dependence of the simulation results in** **Figure 5** ***B*** **.** The control ^14^C-Phe incorporations were obtained with all model parameters at their default values in Table 1, using [*R*]_t_ = 0.5 µM, [EF2]_t_ = 0.6 µM and the added amount of exogenous equal to 0.6 µM. Then simulation sensitivity was evaluated by varying each rate constant from a quarter to tenfold of its default value (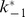 varied to 20 folds) while holding all other parameters unchanged. Vertical bars represent ranges of changes in response to ±5% deviations of [*R*]_t_ from its control level.(TIFF)

Figure S8 **Sensitivity of the half-time gradient with model parameters in Case 2.** The half time *t*_1/2_ is defined as the time taken for the overall rate of protein synthesis to decline to half of its initial rate. The differential change of the half time resulting from 5% increases in [EF2]_t_, i.e., Δ*t*_1/2/_Δ[EF2]_t_, was evaluated from 5% increases in one single parameter while holding the rest at their default values. Vertical bars represent ranges of changes in response to ±5% deviations of [*R*]_t_ from its control level.
(TIFF)

Figure S9 **Only the inhibition modes that minimize ADPR•EF2-ribosome interactions yield a latent dose regime in the toxin-dose response curves.** Cumulative ^14^C-Phe incorporations are recorded 40 min after addition of a bolus ^14^C-Phe-tRNA (4 mM) and toxins (assumed to be the catalytically active fragments capable of inactivating native EF2 immediately upon addition) in varying concentrations into a cell-free system made up of [*R*]_t_ = 0.5 µM and [EF2]_t_ = 6 µM (ten-fold increase). The ^14^C-Phe incorporations in the absence of toxins are taken as 100%.
(TIFF)

Figure S10 **Sensitivity of the double reciprocal slopes on model parameters in Case 5.** Simulation methodology is as described in Figure 9. The slope from the linear portion of the double reciprocal plot is normalized by the corresponding slope of the control case (without ADPR•EF2), and investigated for each inhibition mode and for each model over a wide range of parameter variations. For parameter sensitivity, we varied each of the investigated parameters to various degrees while holding all others constant. Vertical bars represent changes in response to ±5% deviations of [*R*]_t_ from its control value.
(TIFF)

Materials S1 The compressed file “codes.rar” contains the numerical codes required for running the toxin intoxication program and the precompiled executable file. The codes automatically generate a post-simulation report file and transform the simulation results into three encapsulated postscript figures.
(RAR)
