# Supplementary figures and images for: Modes of Action of ADP-Ribosylated Elongation Factor 2 in Inhibiting the Polypeptide Elongation Cycle: A Modeling Study
Source: PLoS One. 2013 Jul 8;8(7):e66446. doi: 10.1371/journal.pone.0066446 (PMC3704607; doi:10.1371/journal.pone.0066446)

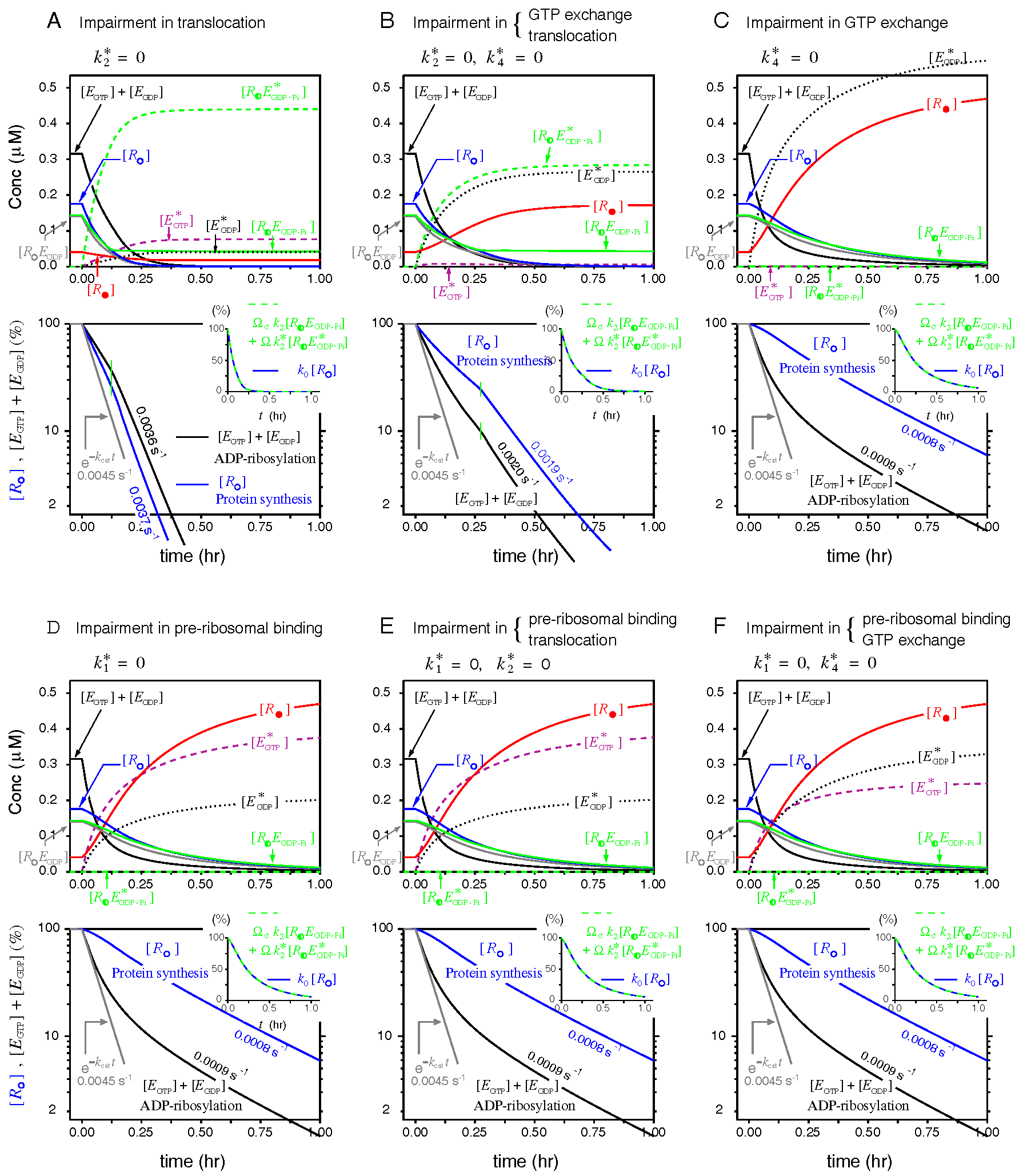

Supplement: Figure S1 — Transient ribosomal phase distributions and inhibition of protein synthesis from model V2 under simulation conditions identical to Figure 4 . The premature turnover rate constant is set as 0.3 s−1. (TIFF) [file pone.0066446.s001.tiff]

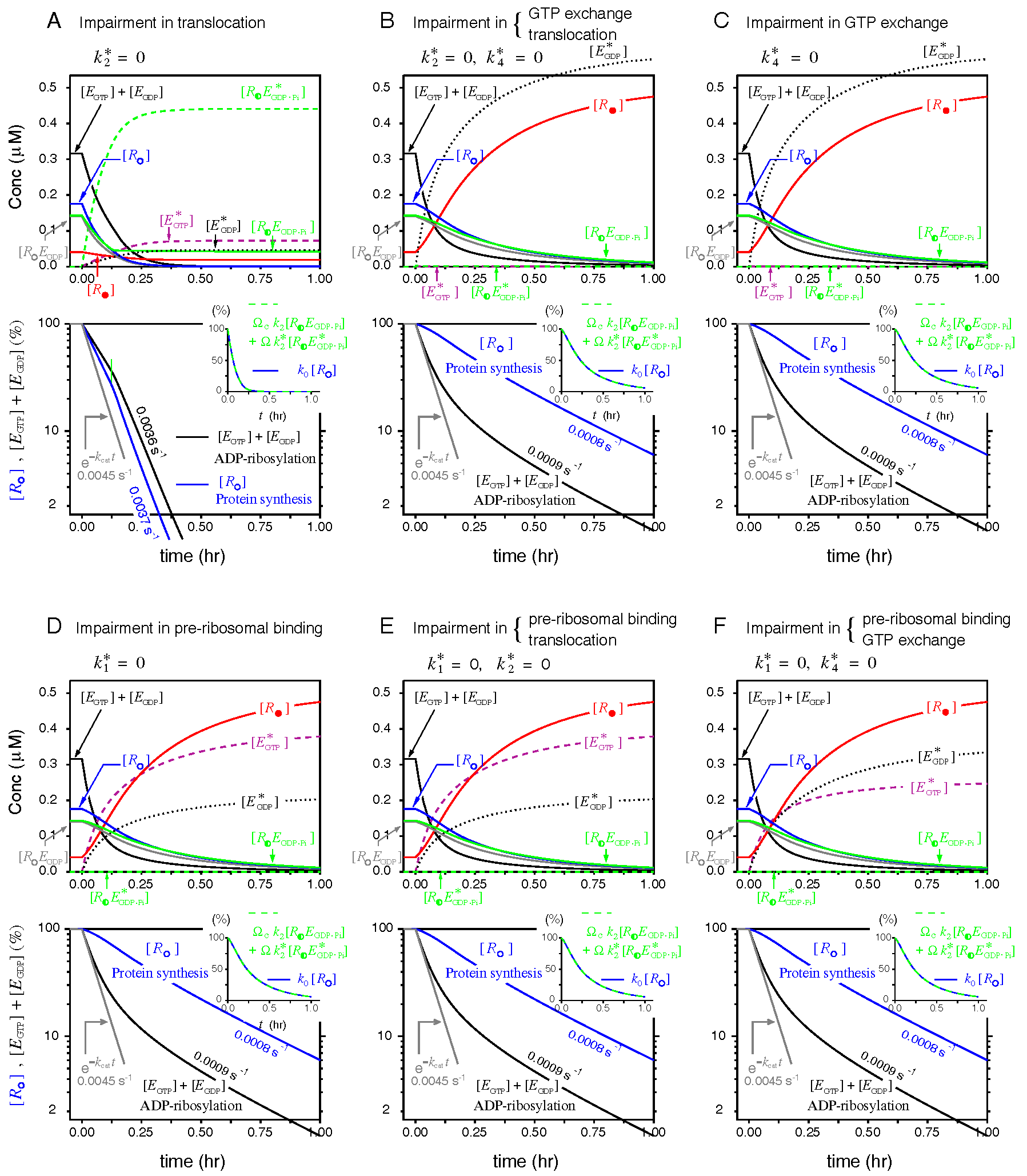

Supplement: Figure S2 — Transient ribosomal phase distributions and inhibition of protein synthesis from model V3 under simulation conditions identical to Figure 4 . The premature turnover rate constant is set as 0.3 s−1. (TIFF) [file pone.0066446.s002.tiff]

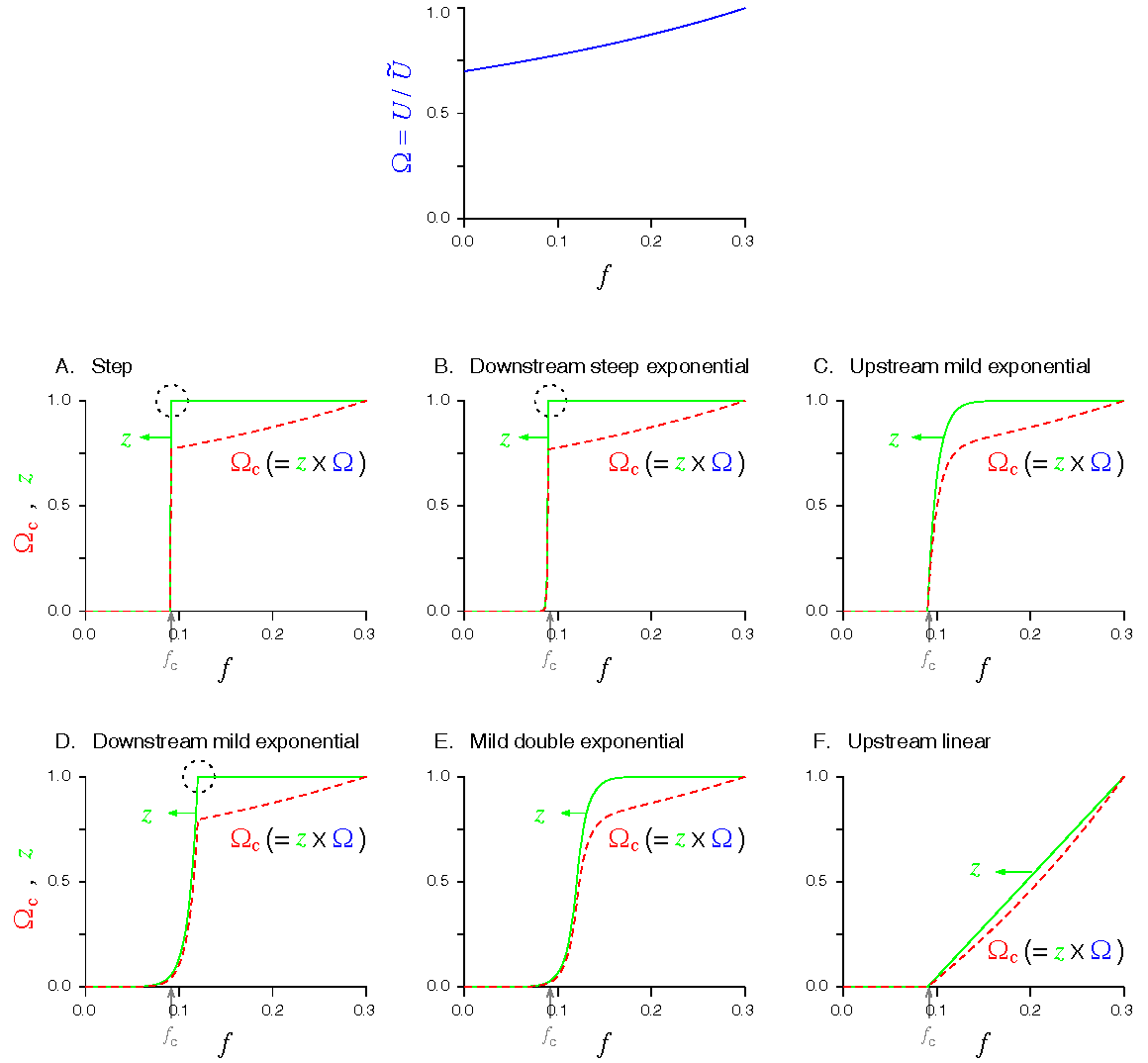

Supplement: Figure S3 — Arbitrary definitions of the permanent stall function Ωc. Assume that Ωc is expressed as the multiplication of z(f) and Ω(f), wherein z(f) is a unit function of f. Six different forms of z(f) are arbitrarily assigned in (A-F), with the common feature that they all monotonically decrease to near zero as f approaches f c ( = 0.09). The tangential discontinuity in z(f) at the f c upstream location is marked by a dashed circle. (TIFF) [file pone.0066446.s003.tiff]

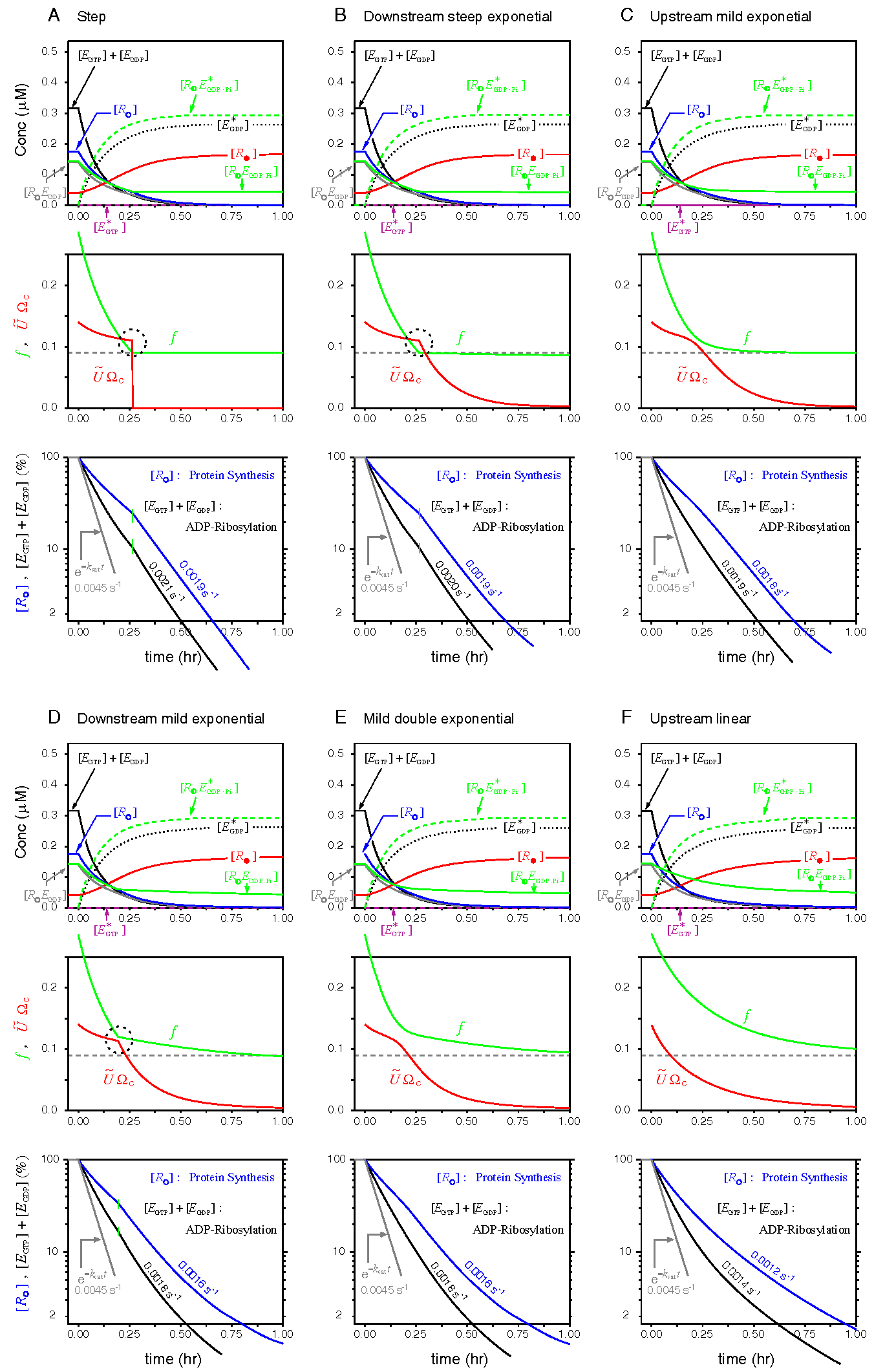

Supplement: Figure S4 — Simulation results under the inhibition mode B of model V1 using the six empirical Ωc defined in Figure S3. Simulation conditions are identical to Figure 4. It is found that the sharp slope change in the inhibition profiles of protein synthesis and native EF2 results from the tangential discontinuity of Ωc at the f c upstream location, not from Ωc approaching zero at f c. (TIFF) [file pone.0066446.s004.tiff]

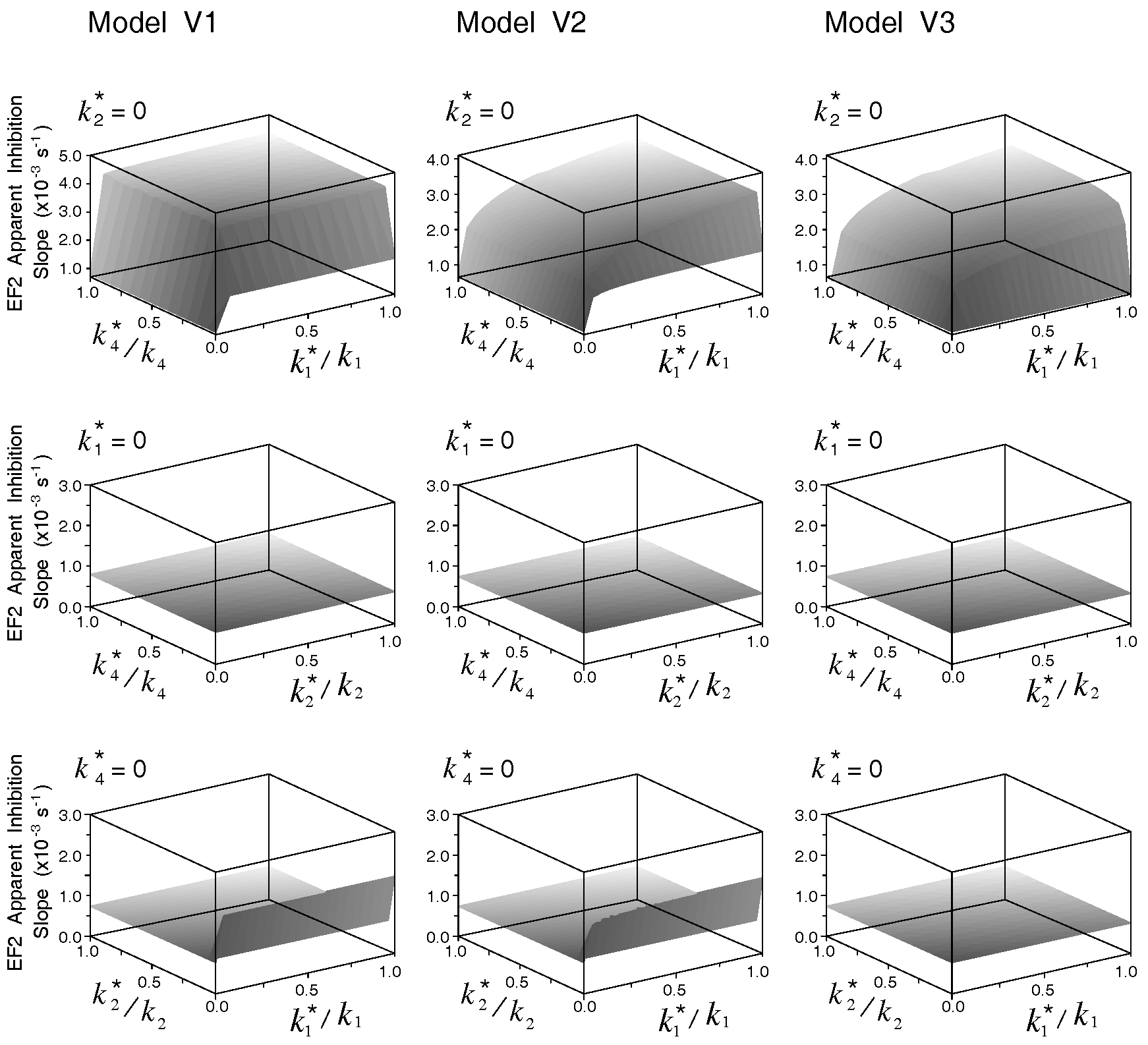

Supplement: Figure S5 — Dependence of the apparent first-order inhibition slope on partial impairment of the ADPR•EF2 parameters. Simulation conditions are identical to Figures S1 and S2 except for the settings of , , and . (TIFF) [file pone.0066446.s005.tiff]

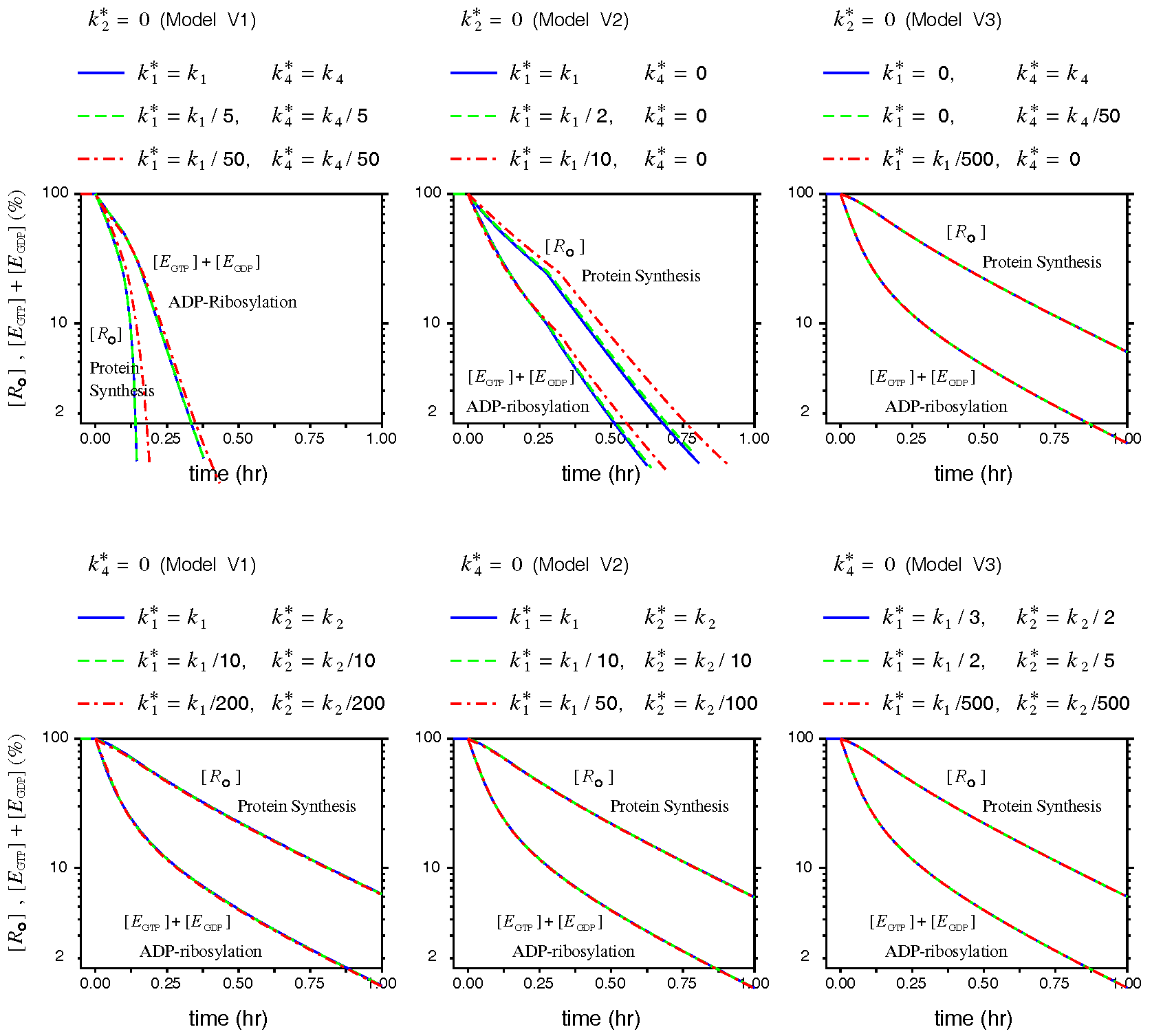

Supplement: Figure S6 — Identical apparent inhibition slopes often yield the same kinetic profiles in the inhibition of protein synthesis and ADP-ribosylation of native EF2. Selection of the ADPR•EF2-related rate constant parameters is based on the simulated slope profiles in Figure S5. (TIFF) [file pone.0066446.s006.tiff]

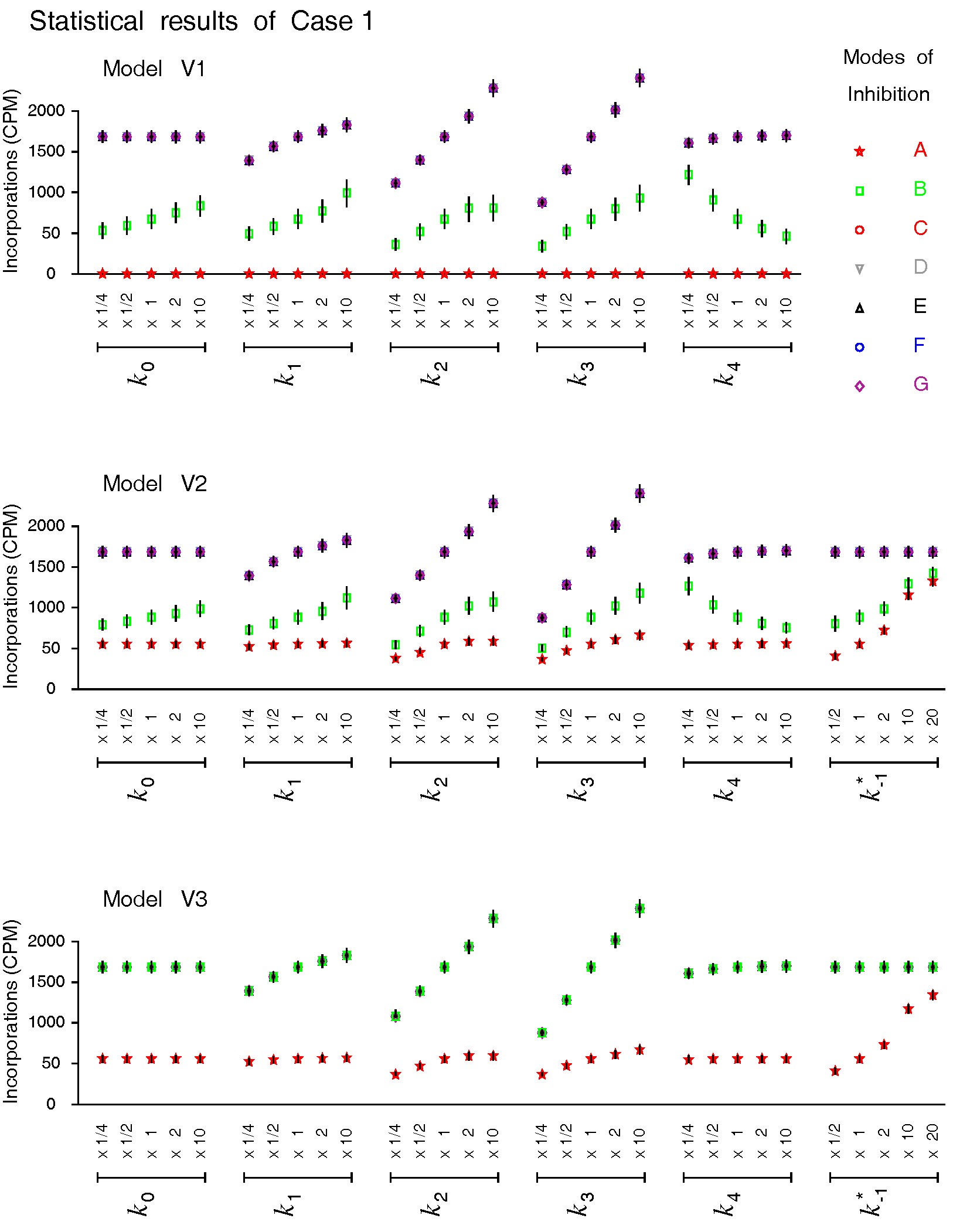

Supplement: Figure S7 — Parameter dependence of the simulation results in Figure 5 B . The control 14C-Phe incorporations were obtained with all model parameters at their default values in Table 1, using [R]t = 0.5 µM, [EF2]t = 0.6 µM and the added amount of exogenous equal to 0.6 µM. Then simulation sensitivity was evaluated by varying each rate constant from a quarter to tenfold of its default value ( varied to 20 folds) while holding all other parameters unchanged. Vertical bars represent ranges of changes in response to ±5% deviations of [R]t from its control level. (TIFF) [file pone.0066446.s007.tiff]

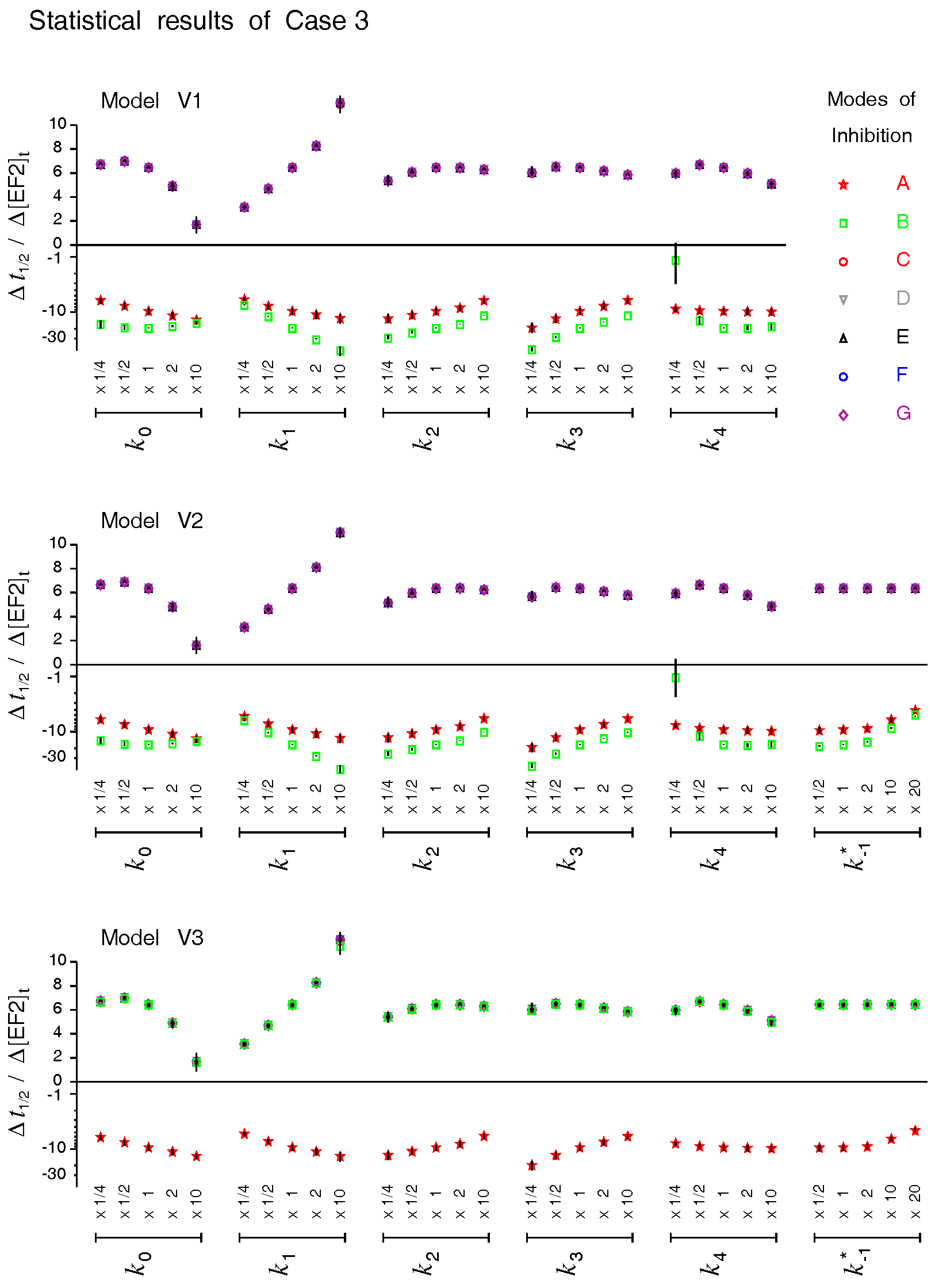

Supplement: Figure S8 — Sensitivity of the half-time gradient with model parameters in Case 2. The half time t 1/2 is defined as the time taken for the overall rate of protein synthesis to decline to half of its initial rate. The differential change of the half time resulting from 5% increases in [EF2]t, i.e., Δt 1/2/Δ[EF2]t, was evaluated from 5% increases in one single parameter while holding the rest at their default values. Vertical bars represent ranges of changes in response to ±5% deviations of [R]t from its control level. (TIFF) [file pone.0066446.s008.tiff]

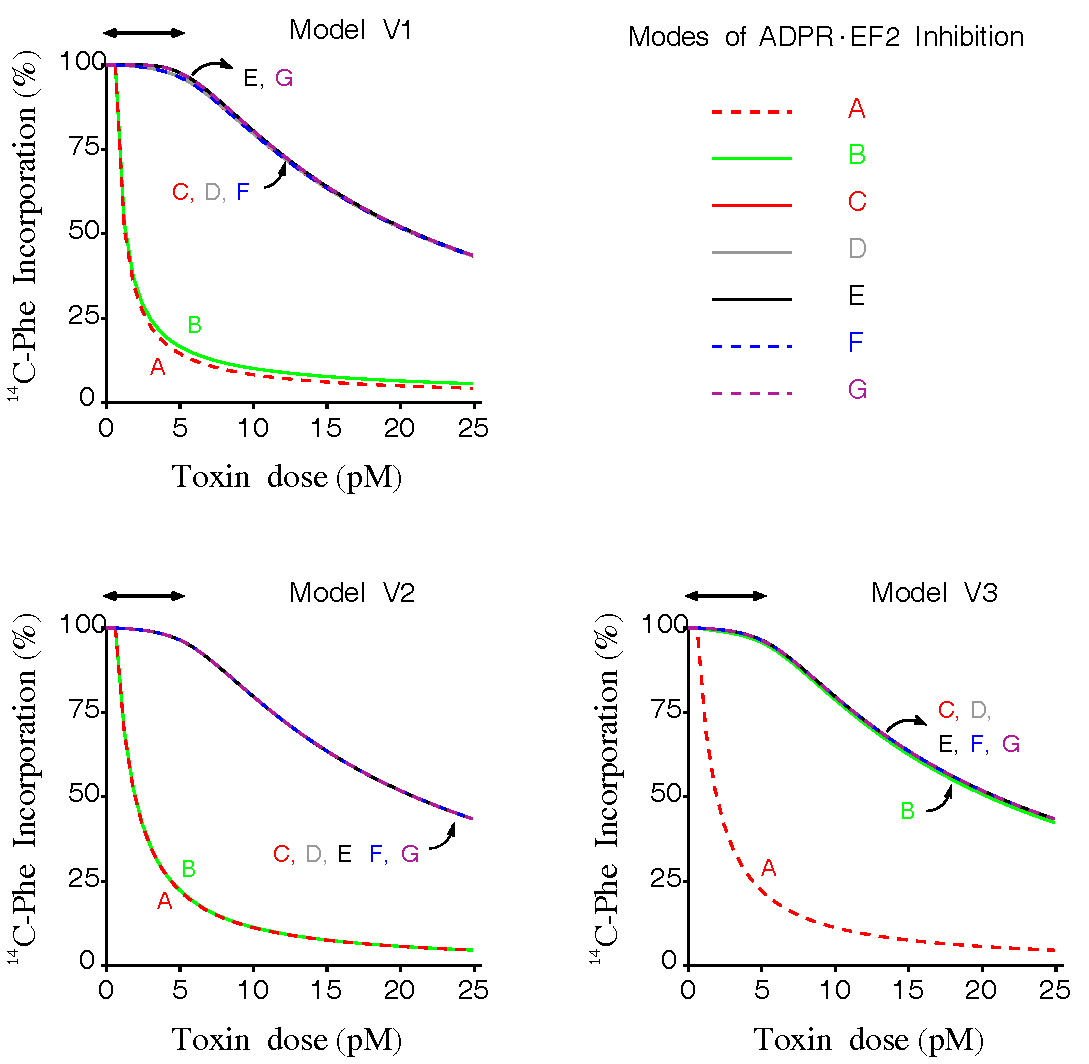

Supplement: Figure S9 — Only the inhibition modes that minimize ADPR•EF2-ribosome interactions yield a latent dose regime in the toxin-dose response curves. Cumulative 14C-Phe incorporations are recorded 40 min after addition of a bolus 14C-Phe-tRNA (4 mM) and toxins (assumed to be the catalytically active fragments capable of inactivating native EF2 immediately upon addition) in varying concentrations into a cell-free system made up of [R]t = 0.5 µM and [EF2]t = 6 µM (ten-fold increase). The 14C-Phe incorporations in the absence of toxins are taken as 100%. (TIFF) [file pone.0066446.s009.tiff]

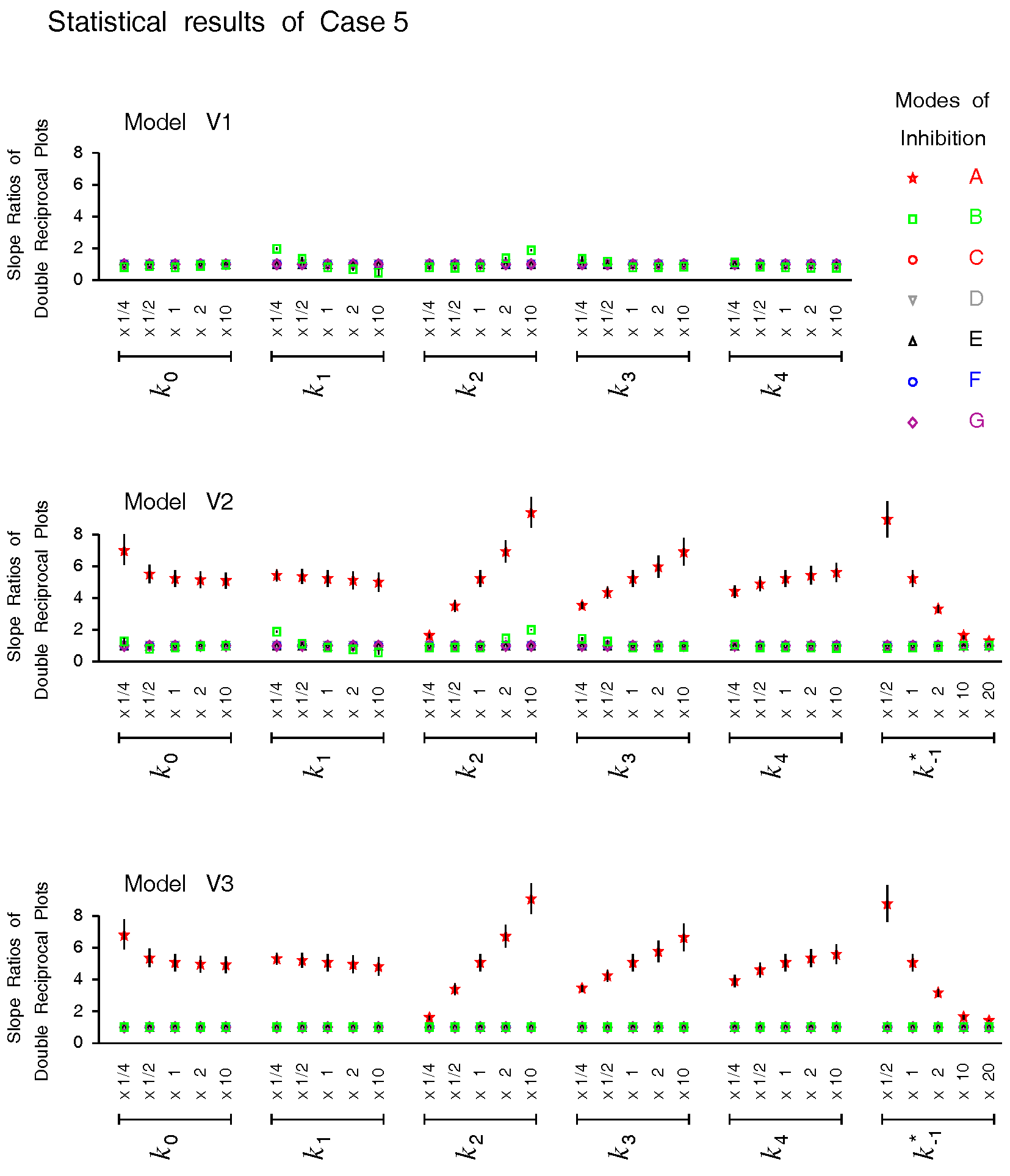

Supplement: Figure S10 — Sensitivity of the double reciprocal slopes on model parameters in Case 5. Simulation methodology is as described in Figure 9. The slope from the linear portion of the double reciprocal plot is normalized by the corresponding slope of the control case (without ADPR•EF2), and investigated for each inhibition mode and for each model over a wide range of parameter variations. For parameter sensitivity, we varied each of the investigated parameters to various degrees while holding all others constant. Vertical bars represent changes in response to ±5% deviations of [R]t from its control value. (TIFF) [file pone.0066446.s010.tiff]
